# Randomized open-label study of second-generation antipsychotics for the treatment of schizophrenia: 104-week final results of the JUMPs study assessing treatment discontinuation, remission, and social functioning

**DOI:** 10.1186/s12888-024-06031-4

**Published:** 2024-09-05

**Authors:** Jun Ishigooka, Kazuyuki Nakagome, Tetsuro Ohmori, Nakao Iwata, Ken Inada, Jun-ichi Iga, Taro Kishi, Kiyoshi Fujita, Yuka Kikuchi, Toshiaki Shichijo, Hideaki Tabuse, Shotatsu Koretsune, Hiroshi Terada, Haruko Terada, Toshifumi Kishimoto, Yuichiro Tsutsumi, Kazutaka Ohi

**Affiliations:** 1 Department of Psychiatry, School of Medicine, Tokyo Women’s Medical University, 8-1, Kawada-cho, Shinjuku-ku, Tokyo, 162-8666 Japan; 2 Department of Psychiatry, National Center of Neurology and Psychiatry, 4 Chome-1-1 Ogawahigashicho, Kodaira, Tokyo, 187-8551 Japan; 3 Department of Psychiatry, Tokushima University Graduate School of Biomedical Sciences, 3-18- 15, Kuramoto-cho, Tokushima, 770-8503 Japan; 4 Department of Psychiatry, Fujita Health University School of Medicine, 1-98 Dengakugakubo, Kutsukake-cho, Toyoake, Aichi 470-1192 Japan; 5 Okehazama Hospital Fujita Kokoro Care Center, Minamiyakata, Sakaecho Toyoake, Aichi 470-1168 Japan; 6 Department of Psychiatry, Akita University Hospital, Hasunuma-44-2 Hiroomote, Akita, 010- 0041 Japan; 7 Mental Clinic Minami, 4 Chome-3-2 Honcho, Iwatsuki Ward, Saitama, 339-0057 Japan; 8 Department of Psychiatry, Holy Cross Hospital, 2431-160 Kujiri, Izumi-cho, Toki-shi, Gifu, 509-5142 Japan; 9 Kokorono Clinic Narimasu, 3-3-15 Narimasu, Itabashi-ku, Tokyo, 175-0094 Japan; 10 Aoi Clinic, 679-1 Okitsunakacho, Shimizu Ward, Shizuoka, 424-0204 Japan; 11 Department of Psychiatry, Nara Medical University, 840 Shijocho, Kashihara, Nara 634-8521 Japan; 12 Department of Psychiatry, Ongata Hospital, 105 Nishiterakatamachi, Hachioji, Tokyo, 192- 0153 Japan; 13 Department of Neuropsychiatry, Kokubu Hospital, 4-672 Asahigaoka, Kashiwara-shi, Osaka 582-0026 Japan; 14 Present address: Institute of CNS Pharmacology, 4-26-11 Sendagaya, Shibuya-ku, Tokyo, 151-0051 Japan; 15 Present address: Department of Psychiatry, Kitasato University School of Medicine, 1-15-1, Kitasato, Minami-ku, Sagamihara-shi, Kanagawa 252-0374 Japan; 16 Present address: Department of Neuropsychiatry, Molecules and Function, Ehime University Graduate School of Medicine, Shitsukawa, Toon, Ehime, 791-0295 Japan; 17 Present address: Heart-Care Clinic Omachi, 1-2-7 Omachi, Akita, 010-0921 Japan; 18 Present address: Akitsu-Kounoike Hospital, 1064 Ikenouchi, Gose-shi, Nara, 639-2273 Japan; 19 Present address: Department of Psychiatry and Psychotherapy, Gifu University Graduate School of Medicine, 1-1 Yanagido, Gifu, 501-1112 Japan

**Keywords:** Discontinuation rate, 104-week effectiveness, Monotherapy, Remission rate, Real-world outcomes, Second-generation antipsychotics

## Abstract

**Background:**

We report the final results of treatment with aripiprazole, blonanserin, and paliperidone from the Japan Useful Medication Program for Schizophrenia (JUMPs), a 104-week naturalistic study.

**Methods:**

JUMPs was an open-label, three-arm, randomized, parallel-group, 104-week study. Patients aged ≥ 20 years with schizophrenia requiring antipsychotic treatment or a switch from previous therapy were enrolled. The primary endpoint was treatment discontinuation rate over 104 weeks. Secondary endpoints included remission rate, Personal and Social Performance (PSP), safety, Positive and Negative Syndrome Scale (PANSS), and quality of life (QOL; EuroQol-5 dimension).

**Results:**

In total, 251 patients received aripiprazole (*n* = 82), blonanserin (*n* = 85), or paliperidone (*n* = 84). Treatment discontinuation rates (aripiprazole, 80.5%; blonanserin, 81.2%; paliperidone, 71.4%) were not significantly different (*p* = 0.2385) among the treatment groups at 104 weeks; comparable outcomes were observed for endpoints, including remission (42.9%, 46.7%, and 45.8%), PANSS, and safety. In the overall cohort, while the improvement in the PSP total score at Week 104 was not significantly different from baseline, a significant improvement (*p* < 0.05) in QOL and total PANSS scores (including all subscales) was observed at Week 104 compared with baseline. Multivariable analysis identified a shorter disease duration and a higher chlorpromazine-equivalent antipsychotic dosage level (≥ 1000 mg) before switching to monotherapy as predictors of treatment discontinuation.

**Conclusions:**

The 104-week treatment outcomes were comparable between groups; the overall trend of improvement in remission rate, safety, and QOL suggests the importance of continued treatment.

**Clinical trial registration:**

UMIN-Clinical Trials Registry UMIN000007942 (public release date: 14/05/2012).

**Supplementary Information:**

The online version contains supplementary material available at 10.1186/s12888-024-06031-4.

## Key points

• The follow-up (104-week) results of the JUMPs study showed that the rate of treatment discontinuation (range: 71–81%) and resolution of symptoms (range: 43–47%) were similar among Japanese patients with schizophrenia treated with aripiprazole, blonanserin, and paliperidone.

• Continued second-generation antipsychotic treatment (104-week) contributes to the maintenance of remission, social functioning, and QOL.

• Treatment discontinuation is likely to get affected by shorter disease duration and higher dose of antipsychotics (chlorpromazine-equivalent antipsychotic dosage level: ≥1000 mg) before switching to single treatment with any of the three second-generation antipsychotics.

**Supplementary Information:**

The online version contains supplementary material available at 10.1186/s12888-024-06031-4.

## Introduction

Schizophrenia is a chronic disease and requires long-term treatment; however, the potential risks and benefits of long-term antipsychotic treatment continue to be debated [1]. Given the chronic nature of schizophrenia, it is becoming increasingly evident that the efficacy results of short-term clinical studies do not provide sufficient evidence to adequately inform the selection of antipsychotics in routine clinical practice [2] to achieve the key treatment aims of symptom remission and recovery, which eventually translate into improved social functioning [3].

To overcome this limitation, studies with a “pragmatic trial” design closely resembling the real-world setting and outcomes important in clinical practice are performed. These large-scale, long-term, naturalistic studies evaluated second-generation antipsychotics (SGAs) in routine clinical practice and included practical endpoints such as discontinuation rates, remission rates, and improvement in the quality of life (QOL) to provide a clear assessment of the long-term effectiveness of a therapy [4–7]. Findings obtained from these studies, along with those from experimental randomized controlled trials (RCTs), are of high importance in real-world treatment practices and health policy decisions [8].

The Japan Useful Medication Program for Schizophrenia (JUMPs) study, a 104-week naturalistic study, compared the effectiveness and safety of three SGAs with flexible oral maintenance dosing (aripiprazole, blonanserin, and paliperidone) in patients with schizophrenia [9]. These three SGAs were selected because they were newly approved in Japan after olanzapine and risperidone, and their discontinuation has not been evaluated in long-term naturalistic RCTs. Among them, data on blonanserin are specific to Asia. Blonanserin was approved in Japan in 2008 as an SGA developed by Sumitomo Pharma, the same company that developed lurasidone (approved just 2 years later in 2010). In contrast to lurasidone, which has been developed as a global drug, blonanserin has been positioned as a local drug in Asia (Japan, South Korea, and China). Blonanserin has a unique affinity profile compared with the standard profile of SGAs, with a higher affinity for dopamine D2 receptors than for serotonin 5-HT2A receptors, but it has been shown to acquire the expected characteristics for SGAs, including a low frequency of extrapyramidal symptoms. It is widely used in countries where it is approved, including Japan. However, to date, there is limited evidence evaluating long-term outcomes of blonanserin treatment in direct comparison with other SGAs [10].

The endpoints used in JUMPs included discontinuation rates, remission rates, and improvements in QOL and social functioning over 104 weeks [9]. Of note, we primarily evaluated the treatment discontinuation for any cause, which had been employed in pivotal preceding studies as a comprehensive indicator that integrates effectiveness, safety, and tolerability as judged by physicians and patients [5–7]. Consequently, the discontinuation and remission rates were selected as endpoints to enable drug selection as a comprehensive measure of treatment effectiveness in clinical practice and highlight standard investigator-patient–based decision-making around treatment. By contrast, other endpoints such as QOL and social functioning are regarded as endpoints of treatment effectiveness in patients with schizophrenia.

The 52-week follow-up results of the pivotal JUMPs study have been reported previously [11]. The primary outcome, namely, the rate of treatment discontinuation at 52 weeks, was similar across the groups: 68.3%, 68.2%, and 65.5% for aripiprazole, blonanserin, and paliperidone, respectively (*p* = 0.9771 for intergroup comparison); the remission rates were 42.3%, 40.7%, and 41.4%, respectively (*p* = 0.9933 for intergroup comparison). Improvements from baseline in social functioning (Personal and Social Performance [PSP] Scale and EuroQol-5 dimension [EQ-5D]) were comparable across the three groups [11].

Herein, we present the final 104-week follow-up results of the JUMPs study and aim to (1) report conclusive evidence from observations such as treatment discontinuation and remission, including findings after 52 weeks of follow-up as surrogate measures of effectiveness and safety to aid in treatment selection in clinical practice, and (2) provide physicians involved in schizophrenia treatment with detailed information on SGAs commonly used in Asian countries, including Japan, obtained using a 104-week naturalistic RCT study design.

## Methods

### Study design

This was an open-label, three-arm, multicenter, randomized, parallel-group study involving Japanese patients with schizophrenia. Eligible patients were randomized (1:1:1 ratio) to receive aripiprazole, blonanserin, or paliperidone as monotherapy. Patients were evaluated at baseline; the start of monotherapy; and 8, 12, 26, 52, 78, and 104 weeks of follow-up. Computer-based randomization was performed by Mebix, Inc., a third-party clinical research organization (Minato-ku, Tokyo; Akasaka Intercity), using the “variable permuted block randomization” method (35 blocks, with each block having sizes of 12 and 15) with no stratification factors. The randomization method is described in detail in a previously published protocol [11]. The study was approved by the institutional review boards at each study site and conducted in accordance with the Declaration of Helsinki, the International Conference on Harmonisation Tripartite Guideline for Good Clinical Practice, and the Ethics Guidelines for Clinical Research [12]. All patients provided written informed consent. The study is registered at UMIN-CTR: https://center6.umin.ac.jp/cgi-open-bin/ctr/ctr_view.cgi?recptno=R000009273.

### Patients

Complete inclusion and exclusion criteria are presented in a previously published protocol [9]. Briefly, outpatients aged ≥ 20 years with schizophrenia diagnosed using the Diagnostic and Statistical Manual of Mental Disorders, Fourth Edition, Text Revision (DSM-IV-TR), who were either treatment naïve or switching from their current antipsychotic medication owing to lack of efficacy, intolerability, or other reasons and capable of providing written consent were enrolled in the study.

### Procedures

During the dose-adjustment period of 4 weeks (maximum 8 weeks), patients requiring a change from prior antipsychotics were switched to one of the three protocol treatments. During the titration/dose-adjustment period, the initial and maximum oral maintenance doses were, respectively, ≤ 12 mg/day and 30 mg/day for aripiprazole, ≤ 8 mg/day and 24 mg/day for blonanserin, and ≤ 6 mg/day and 12 mg/day once daily after breakfast for paliperidone. After the dose-adjustment period, and depending on the patients’ clinical condition, the dosage of each study drug could be adjusted within the approved dosage range during the study period (aripiprazole 12–30 mg/day, blonanserin 8–24 mg/day, and paliperidone 6–12 mg/day). The chlorpromazine (CP)-equivalent dose (dose equivalence of antipsychotics [2012 version]) [13] was evaluated from the number of prior antipsychotic medications taken before initiating the study treatment and the total doses of the study medication used. Details of concomitant medication used are presented in Online Resource 1 Table S1.

The physician inquired about and assessed treatment compliance at each visit. Any changes in medication due to inadequate compliance were made at the physician’s discretion.

### Outcome measurements

The primary endpoint was the rate of discontinuation due to any cause, evaluated at 104 weeks. Secondary endpoints included remission rate (Andreasen definition: rating below mild on all eight items of the Positive and Negative Syndrome Scale [PANSS] for ≥ 6 consecutive weeks) [14], social activity score (PSP Scale [15] and EQ-5D [16]), alleviation of psychiatric symptoms assessed using the PANSS score, Clinical Global Impression-Improvement (CGI-I) score [17], and Clinical Global Impression-Suicide Scale (CGI-SS) score [18]. Other secondary endpoints included the incidence of adverse events (AEs) and drug-induced extrapyramidal symptom rating scale (DIEPSS) scores [19]. Serious adverse drug reactions (serious ADRs) were AEs that were judged by physicians to be associated with the study treatment and to be serious symptoms and were also set as secondary endpoints. Additionally, the remission rate was calculated at Week 52 among patients who completed the 104-week follow-up; changes in PSP, EQ-5D, PANSS, and DIEPSS scores at Week 104 from Week 52 were evaluated.

### Statistical analysis

The intent-to-treat (ITT) population was defined as all randomized patients who received the study medication. The per-protocol set was defined as patients in the ITT population without significant protocol relevant deviations determined by the Steering Committee; the safety population comprised all patients with available safety data, i.e., the presence/absence of AEs or DIEPSS. The primary endpoint analysis of treatment discontinuation was estimated using the Kaplan-Meier (KM) method, along with the corresponding 95% confidence intervals (CIs). The Kruskal-Wallis test and one-way analysis of variance were used for determining significant differences on a continuous dependent variable by a categorical independent variable (with two or more groups). The paired *t*-test was used to compare the change from baseline in each treatment group, and the chi-square test was used to compare treatment groups. In the exploratory analysis, to identify other factors that may affect the continuity of treatment, Cox proportional hazards regression models were used to evaluate the risk factors for treatment discontinuation and determine the hazard ratios (HRs) and corresponding 95% CIs. Univariate and multivariable Cox proportional hazards regression analyses were conducted using variables such as baseline CP-equivalent dose ≥ 1000 mg, adjusted for sex (female), duration of illness (≥ 6 years), and PANSS total scores (≥ 80) at baseline. The cutoff point of 1000-mg CP-equivalent dose was considered because the recommended standard daily dose of antipsychotic drugs is less than this dose [20]. Furthermore, according to the critical period, the duration of illness of 6 years was determined [21] and a PANSS score of 80 was set between “moderately ill” and “markedly ill” [22]. Statistical analysis was contracted to Mebix (Minato-ku, Tokyo; Akasaka Intercity) and performed using SAS version 9.4 (SAS Institute Inc., Cary, NC); a *p* value of < 0.05 was considered to dte statistical significance for all tests.

## Results

**Fig. 1:**
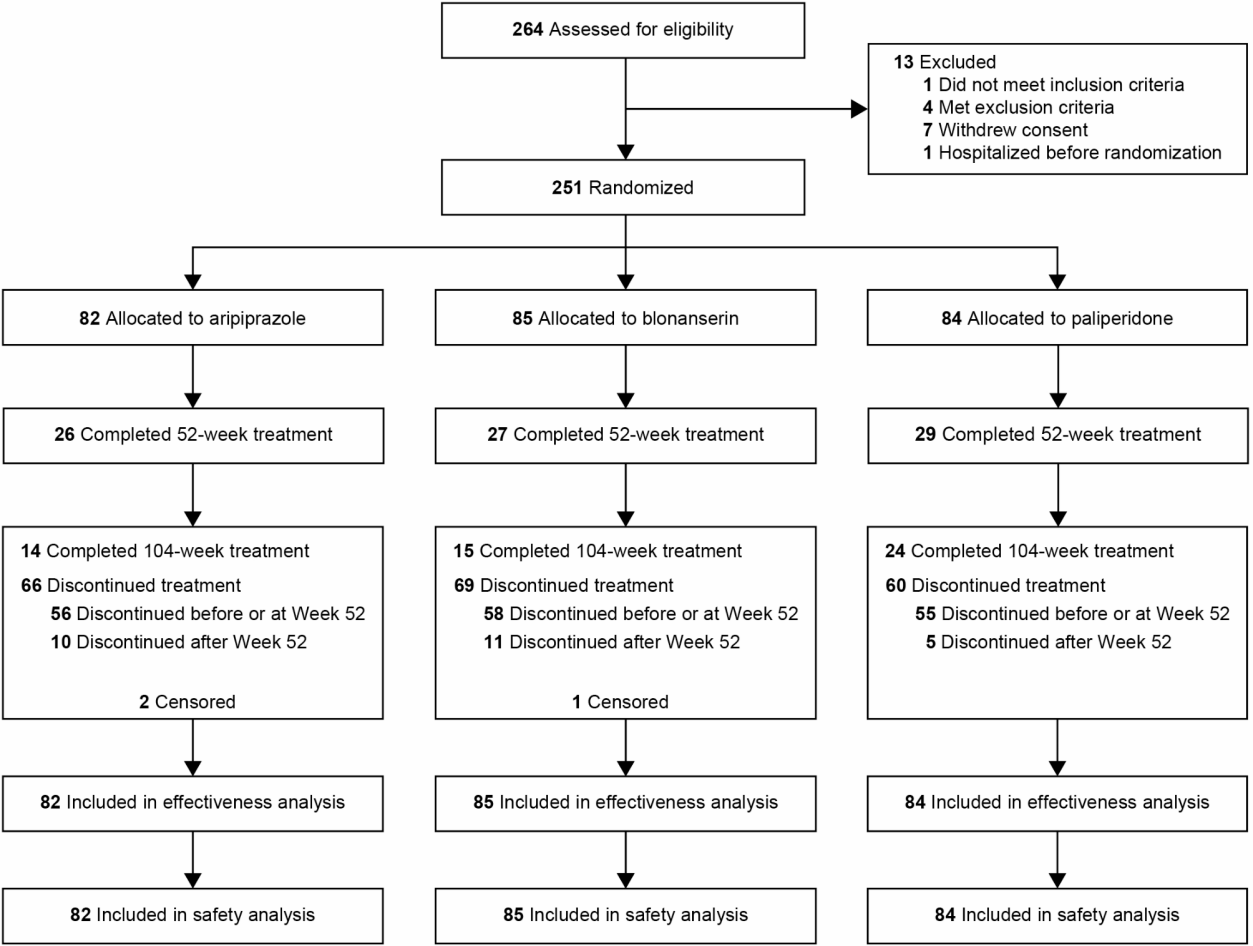
Patient disposition

A total of 251 patients were randomized in a 1:1:1 ratio to receive aripiprazole (*n* = 82), blonanserin (*n* = 85), or paliperidone (*n* = 84), of whom 26, 27, and 29 patients completed the 52-week follow-up, and 14, 15, and 24 patients completed the 104-week follow-up periods, respectively (Fig. 1). Overall, patient demographics and baseline characteristics were similar among the three groups (Table 1).

**Table 1:** Patient demographics and baseline characteristics (ITT cohort)

Characteristic, mean (SD)	Overall *N* = 251	Aripiprazole group *n* = 82	Blonanserin group *n* = 85	Paliperidone group *n* = 84
Age, years	46.5 (13.3)	48.0 (14.1)	46.7 (12.6)	45.0 (13.2)
Sex, male, *n* (%)	133 (53.0)	42 (51.2)	48 (56.5)	43 (51.2)
Height, cm	162.9 (9.0)	161.5 (9.6)	163.7 (9.0)	163.4 (8.1)
Body weight, kg	67.7 (14.9)	66.5 (17.4)	69.1 (14.5)	67.3 (12.5)
Disease type (DSM-IV classifications), *n* (%)				
Paranoid	161 (64.1)	55 (67.1)	51 (60.0)	55 (65.5)
Disorganized	15 (6.0)	3 (3.7)	6 (7.1)	6 (7.1)
Catatonic	9 (3.6)	5 (6.1)	2 (2.4)	2 (2.4)
Undifferentiated	22 (8.8)	5 (6.1)	7 (8.2)	10 (11.9)
Residual	44 (17.5)	14 (17.1)	19 (22.4)	11 (13.1)
Clinical picture (selected parameters), *n* (%)				
Hallucinatory/delusion state	76 (30.3)	28 (34.1)	25 (29.4)	23 (27.4)
Delusions in foreground	27 (10.8)	10 (12.2)	9 (10.6)	8 (9.5)
Loss of initiative/apathy in foreground I	14 (5.6)	4 (4.9)	4 (4.7)	6 (7.1)
Loss of initiative/apathy in foreground II	123 (49.0)	42 (51.2)	44 (51.8)	37 (44.0)
Neurosis-like state in foreground	16 (6.4)	3 (3.7)	4 (4.7)	9 (10.7)
Disease duration, years	17.1 (12.3)	18.9 (13.3)	16.5 (11.7)	15.8 (11.9)
< 6, *n* (%)	57 (22.7)	19 (23.2)	18 (21.2)	20 (23.8)
≥ 6, *n* (%)	194 (77.3)	63 (76.8)	67 (78.8)	64 (76.2)
Prior treatment, *n* (%)				
No	15 (6.0)	5 (6.1)	4 (4.7)	6 (7.1)
Yes	236 (94.0)	77 (93.9)	81 (95.3)	78 (92.9)
Reason for switching from prior medications, *n* (%)				
Lack of efficacy of prior medications	99 (41.9)	32 (41.6)	34 (42.0)	33 (42.3)
Treated with polypharmacy	23 (9.7)	8 (10.4)	10 (12.3)	5 (6.4)
Lowered tolerability	54 (22.9)	19 (24.7)	13 (16.0)	22 (28.2)
Patient’s reason	30 (12.7)	7 (9.1)	11 (13.6)	12 (15.4)
Others	30 (12.7)	11 (14.3)	13 (16.0)	6 (7.7)
Monotherapy/polypharmacy, *n* (%)				
Monotherapy (1)	182 (77.1)	53 (68.8)	62 (76.5)	67 (85.9)
Polypharmacy (≥ 2)	54 (22.9)	24 (31.2)	19 (23.5)	11 (14.1)
Chlorpromazine-equivalent dose, mean (SD) and *n* (%)				
Mean (SD)	442.5 (321.7)	453.6 (335.1)	436.4 (304.0)	438.0 (329.8)
< 1000 mg	221 (93.6)	71 (92.2)	76 (93.8)	74 (94.9)
≥ 1000 mg	15 (6.4)	6 (7.8)	5 (6.2)	4 (5.1)
< 400 mg	116 (49.2)	38 (49.4)	39 (48.1)	39 (50.0)
≥ 400 mg	120 (50.8)	39 (50.6)	42 (51.9)	39 (50.0)
Comorbidities, *n* (%)	167 (66.5)	63 (76.8)	52 (61.2)	52 (61.9)
Hypertension	29 (11.6)	13 (15.9)	9 (10.6)	7 (8.3)
Diabetes	24 (9.6)	9 (11.0)	8 (9.4)	7 (8.3)
Hyperlipidemia	38 (15.1)	14 (17.1)	13 (15.3)	11 (13.1)
Others	142 (56.6)	55 (67.1)	45 (52.9)	42 (50.0)
Social functioning (PSP) total score	56.3 (20.8)	59.0 (21.0)	53.6 (21.1)	56.5 (20.4)
Social functioning (EQ-5D utility value)	0.788 (0.167)^a^	0.779 (0.168)	0.768 (0.176)	0.818 (0.153)^b^
PANSS total score	73.8 (21.1)	75.2 (22.2)	75.7 (18.4)	70.5 (22.4)
DIEPSS overall severity, *n* (%)				
None, normal	163 (64.9)	44 (53.7)	60 (70.6)	59 (70.2)
Minimal, questionable	60 (23.9)	24 (29.3)	17 (20.0)	19 (22.6)
Mild	20 (8.0)	8 (9.8)	7 (8.2)	5 (6.0)
Moderate	7 (2.8)	5 (6.1)	1 (1.2)	1 (1.2)
Severe	1 (0.4)	1 (1.2)	0 (0.0)	0 (0.0)
Per-protocol switch to monotherapy within 8 weeks from randomization, *n* (%)	224 (89.2)	75 (91.5)	76 (89.4)	73 (86.9)
Days elapsed until switch to monotherapy^c^	20.0 (19.0)^d^	20.4 (19.5)^e^	20.5 (20.5)^f^	19.2 (17.2)^g^
Days elapsed until switch to monotherapy among patients with prior medication	21.3 (19.0)^h^	21.5 (19.5)^i^	21.3 (20.5)^g^	20.9 (16.9)^j^

*DIEPSS* Drug-Induced Extrapyramidal Symptoms Scale, *DSM* Diagnostic and Statistical Manual of Mental Disorders, *EQ-5D* EuroQol-5 dimension, *ITT* intent-to-treat, *PANSS* Positive and Negative Syndrome Scale, *PSP* Personal and Social Performance Scale, *SD* standard deviation

^a^*n* = 250; ^b^*n* = 83; ^c^elapsed days for patients without prior medication were defined as zero; ^d^*n* = 224; ^e^*n* = 75; ^f^*n* = 76; ^g^*n* = 73; ^h^*n* = 210; ^i^*n* = 70; ^j^*n* = 67

**Fig. 2:**
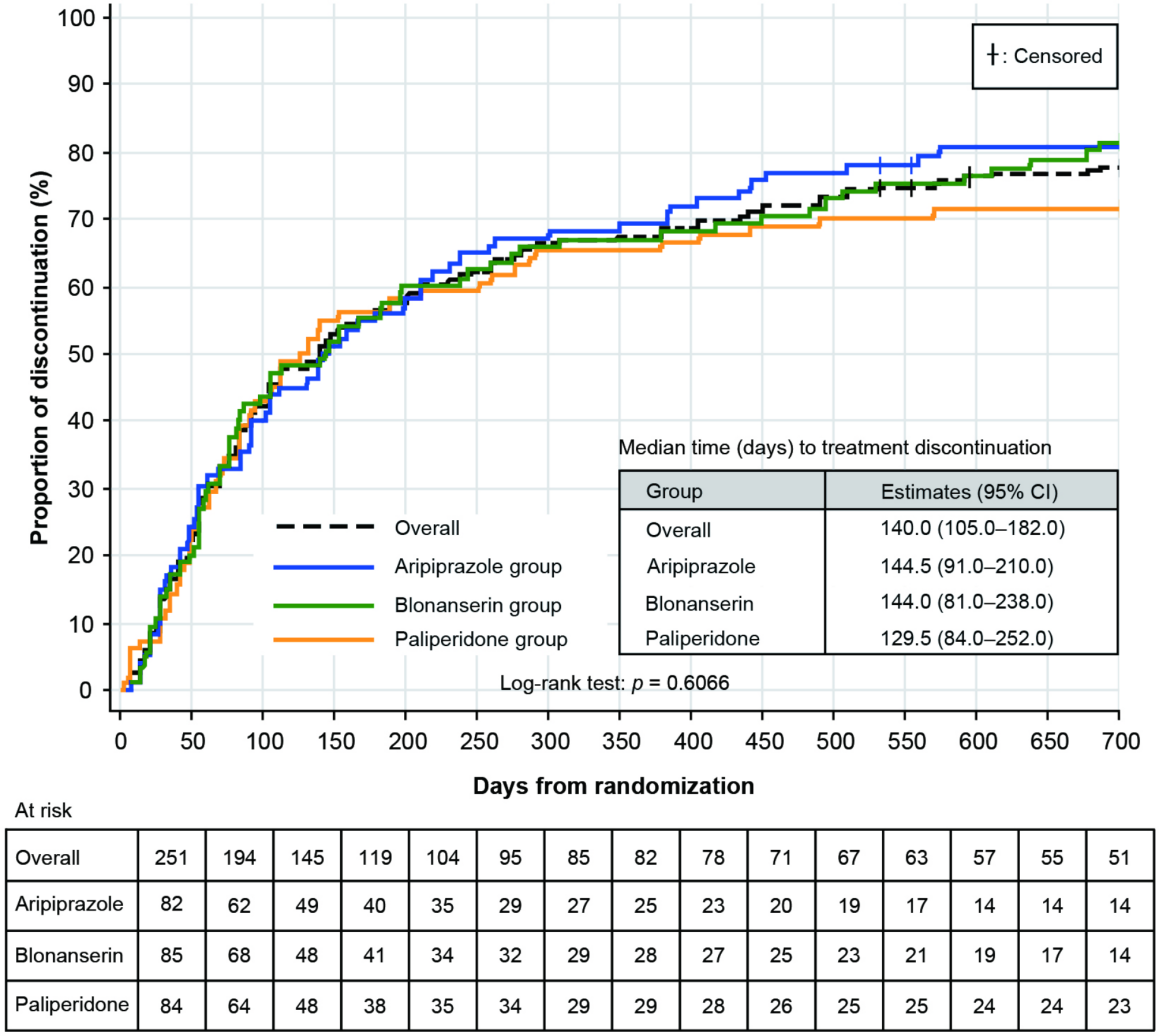
Proportion of patients who had treatment discontinuation over Week 104 (primary endpoint). *CI* confidence interval

The primary analyses were performed on the ITT and safety populations; however, both comprised the same patient population, as there were no cases of protocol deviations or missing safety data. Before 104 weeks, the overall discontinuation rate was 67.3% over 52 weeks (aripiprazole [68.3%], blonanserin [68.2%], and paliperidone [65.5%]), with no significant difference among the treatment groups (*p* = 0.9060; by KM analysis, log-rank test, *p* = 0.9771). The final treatment discontinuation rates increased from 52 weeks to 104 weeks (aripiprazole [80.5% {66/82 patients}], blonanserin [81.2% {69/85 patients}], and paliperidone [71.4% {60/84 patients}]), with no significant differences among the treatment groups (χ^2^ test, *p* = 0.2385; Online Resource 1 Table S2; by KM analysis, log-rank test, *p* = 0.6066 [Fig. 2]). The median (95% CI) time to treatment discontinuation was 144.5 (91.0–210.0), 144.0 (81.0–238.0), and 129.5 (84.0–252.0) days in the aripiprazole, blonanserin, and paliperidone groups, respectively (Fig. 2).

Overall, treatment withdrawals due to inadequate efficacy increased from 29.9% at Week 52 to 34.3% at Week 104. Treatment withdrawals due to inadequate efficacy occurred in 34.1%, 32.9%, and 22.6% of patients at Week 52 and increased to 41.5%, 35.3%, and 26.2% at Week 104 in the aripiprazole, blonanserin, and paliperidone groups, respectively; these rates due to inadequate efficacy were higher for the aripiprazole and blonanserin groups than for the paliperidone group (Online Resource 1 Table S2).

Overall discontinuations due to poor tolerability or AEs, based on physician judgment, increased from 19.1% at Week 52 to 20.3% at Week 104. Discontinuations due to poor tolerability or AEs occurred in 19.5%, 12.9%, and 25.0% of patients at Week 52 and in 19.5%, 15.3%, and 26.2% of patients at Week 104 in the aripiprazole, blonanserin, and paliperidone groups, respectively; the discontinuation rate due to poor tolerability or AEs was higher for paliperidone than for aripiprazole and blonanserin (Online Resource 1 Table S2). Overall treatment withdrawals based on patient request increased from 11.2% at Week 52 to 12.7% at Week 104. Treatment withdrawals due to patient request occurred in 8.5% and 14.1% of patients at Week 52 and increased to 11.0% and 16.5% at Week 104 in the aripiprazole and blonanserin groups, respectively; the discontinuation rate did not increase for the paliperidone group, which was 10.7% at Week 52 and Week 104 (Online Resource 1 Table S2).

Univariate analysis did not reveal any factors significantly associated with treatment discontinuation (Table 2). Multivariable analysis showed that discontinuation was positively affected by prior medication of a ≥ 1000-mg CP-equivalent dose (HR, 1.826; 95% CI, 1.042–3.199; *p* = 0.0353) and negatively affected by disease duration ≥ 6 years (HR, 0.692; 95% CI, 0.488–0.981; *p* = 0.0387; Table 2).

**Table 2:** Investigation of factors affecting treatment discontinuation rate (Cox proportional hazards modeling)

Factor		Univariate			Multivariable		
		HR	95% CI	*p* value	HR	95% CI	*p* value
CP-equivalent dose	≥ 1000 mg	1.624	0.940–2.806	0.0820	1.826	1.042–3.199	0.0353
Sex	Female	1.135	0.851–1.514	0.3882	1.164	0.872–1.555	0.3022
Duration of disease	≥ 6 years	0.740	0.528–1.037	0.0806	0.692	0.488–0.981	0.0387
PANSS total score	≥ 80	0.984	0.729–1.329	0.9151	1.055	0.776–1.436	0.7313

*CI* confidence interval, *CP* chlorpromazine, *HR* hazard ratio, *PANSS* Positive and Negative Syndrome Scale

A gradual increase was noted in the percentage of patients achieving remission over 104 weeks, which did not differ significantly between the treatment groups (Table 3). In each group, the remission rate reached 41–42% (aripiprazole, 42.3%; blonanserin, 40.7%; paliperidone, 41.4%) at 52 weeks and remained largely stable among those who continued treatment until Week 104 (aripiprazole, 42.9%; blonanserin, 46.7%; paliperidone, 45.8%; Table 3).

**Table 3:** Proportion of remission

Time point		All groups			Aripiprazole group			Blonanserin group			Paliperidone group			χ^2^ test (*p* value)
		Total (*N*)	Remission rate		Total (*n*)	Remission rate		Total (*n*)	Remission rate		Total (*n*)	Remission rate		
			*n* (%)	95% CI		*n* (%)	95% CI		*n* (%)	95% CI		*n* (%)	95% CI	
Before the start of the study treatment		251	74 (29.5)	23.9–35.5	82	23 (28.0)	18.7–39.1	85	23 (27.1)	18.0–37.8	84	28 (33.3)	23.4–44.5	0.6312
Initiation of single agent		224	61 (27.2)	21.5–33.6	75	23 (30.7)	20.5–42.4	76	18 (23.7)	14.7–34.8	73	20 (27.4)	17.6–39.1	0.6281
Week 8		186	37 (19.9)	14.4–26.4	62	14 (22.6)	12.9–35.0	62	10 (16.1)	8.0–27.7	62	13 (21.0)	11.7–33.2	0.6449
Week 12		157	37 (23.6)	17.2–31.0	53	13 (24.5)	13.8–38.3	50	11 (22.0)	11.5–36.0	54	13 (24.1)	13.5–37.6	0.9498
Week 26		112	31 (27.7)	19.6–36.9	38	11 (28.9)	15.4–45.9	37	7 (18.9)	8.0–35.2	37	13 (35.1)	20.2–52.5	0.2899
Week 52	All patients evaluated	82	34 (41.5)	30.7–52.9	26	11 (42.3)	23.4–63.1	27	11 (40.7)	22.4–61.2	29	12 (41.4)	23.5–61.1	0.9933
	^a^Only for 104-week patients	53	23 (43.4)	29.8–57.7	14	6 (42.9)	17.7–71.1	15	6 (40.0)	16.3–67.7	24	11 (45.8)	25.6–67.2	0.9370
Week 104		53	24 (45.3)	31.6–59.6	14	6 (42.9)	17.7–71.1	15	7 (46.7)	21.3–73.4	24	11 (45.8)	25.6–67.2	0.9764

*CI* confidence interval

^a^Remission rates were calculated for patients who completed 104 weeks of continuation to compare the proportion at 104 weeks

The personal and social functioning (as measured using the PSP) total score significantly improved up to 52 weeks, but the improvement was not significant at Week 104 in the overall treatment groups. The change in PSP at Week 104 from Week 52 was also not significant. The improvement in PSP was not significantly different between the groups at 104 weeks (Table 4).

**Table 4:** Social functioning and QOL score (determined by the PSP and EQ-5D)

Item	Treatment group		Baseline	Week 26		Week 52		Week 104		
				Score	Change from baseline	Score	Change from baseline	Score	Change from baseline at Week 104	Change from 52-week score
PSP	All groups	*N*	251	105	105	77	77	52	52	50
Total score		Mean (SD)	56.3 (20.8)	61.5 (21.5)	5.4 (16.8)*	61.4 (21.8)	7.8 (17.7)*	59.8 (24.1)	4.1 (18.3)	−3.6 (16.5)
		Median	60.0	65.0	0.0	65.0	0.0	65.0	0.0	0.0
		Min, max	6, 100	4, 95	−71, 73	6, 95	−25, 74	6, 98	−56, 55	−74, 18
	Aripiprazole group	*n*	82	34	34	23	23	14	14	12
		Mean (SD)	59.0 (21.0)	67.5 (21.7)	6.4 (15.7)*	61.9 (25.8)	5.9 (14.4)	64.0 (27.8)	8.3 (16.9)	1.8 (9.6)
		Median	65.0	68.0	0.0	70.0	0.0	67.5	3.0	0.0
		Min, max	6, 100	11, 95	−21, 56	6, 95	−19, 42	6, 96	−15, 48	−15, 18
	Blonanserin group	*n*	85	37	37	27	27	15	15	15
		Mean (SD)	53.6 (21.1)	60.0 (18.8)	7.8 (16.9)*	59.9 (23.2)	9.5 (18.7)*	55.5 (25.7)	1.5 (27.6)	−8.8 (20.8)
		Median	60.0	65.0	1.0	65.0	1.0	61.0	0.0	0.0
		Min, max	7, 85	10, 90	−25, 73	6, 95	−25, 66	9, 98	−56, 55	−61, 10
	Paliperidone group	*n*	84	34	34	27	27	23	23	23
		Mean (SD)	56.5 (20.4)	57.3 (23.3)	1.9 (17.5)	62.4 (16.7)	7.7 (19.5)	60.1 (21.0)	3.1 (10.4)	−3.1 (15.8)
		Median	60.0	60.0	0.0	65.0	0.0	65.0	0.0	0.0
		Min, max	6, 95	4, 95	−71, 52	20, 95	−7, 74	6, 95	−8, 40	−74, 11
EQ-5D	All groups	*n*	250	106	106	79	79	51	51	51
(utility value)		Mean (SD)	0.788 (0.167)	0.834 (0.171)	0.042 (0.166)*	0.842 (0.164)	0.048 (0.147)*	0.893 (0.156)	0.059 (0.179)*	0.002 (0.130)
		Median	0.771	0.786	0.000	0.804	0.000	1.000	0.000	0.000
		Min, max	0.18, 1.00	0.12, 1.00	−0.35, 0.52	0.41, 1.00	−0.31, 0.53	0.47, 1.00	−0.31, 0.52	−0.31, 0.30
	Aripiprazole group	*n*	82	34	34	25	25	14	14	14
		Mean (SD)	0.779 (0.168)	0.829 (0.174)	0.043 (0.198)	0.831 (0.161)	0.066 (0.179)	0.867 (0.165)	0.074 (0.210)	−0.011 (0.160)
		Median	0.771	0.786	0.000	0.768	0.000	1.000	0.032	0.000
		Min, max	0.39, 1.00	0.47, 1.00	−0.34, 0.47	0.59, 1.00	−0.31, 0.53	0.59, 1.00	−0.31, 0.39	−0.31, 0.26
	Blonanserin group	*n*	85	36	36	27	27	15	15	15
		Mean (SD)	0.768 (0.176)	0.812 (0.189)	0.048 (0.133)*	0.814 (0.170)	0.046 (0.133)	0.830 (0.182)	0.046 (0.181)	−0.017 (0.135)
		Median	0.768	0.786	0.000	0.786	0.000	0.804	0.000	0.000
		Min, max	0.18, 1.00	0.12, 1.00	−0.23, 0.34	0.41, 1.00	−0.25, 0.34	0.47, 1.00	−0.23, 0.34	−0.30, 0.26
	Paliperidone group	*n*	83	36	36	27	27	22	22	22
		Mean (SD)	0.818 (0.153)	0.862 (0.148)	0.034 (0.166)	0.879 (0.160)	0.033 (0.130)	0.952 (0.111)	0.059 (0.164)	0.023 (0.107)
		Median	0.786	0.902	0.000	1.000	0.000	1.000	0.000	0.000
		Min, max	0.48, 1.00	0.59, 1.00	−0.35, 0.52	0.48, 1.00	−0.30, 0.29	0.59, 1.00	−0.21, 0.52	−0.21, 0.30

There was no significant difference over 104 weeks between the groups (assessed using analysis of variance)

*EQ-5D* EuroQol-5 Dimension, *max* maximum, *min* minimum, *PSP* Personal and Social Performance Scale, *QOL* quality of life, *SD* standard deviation

*p < 0.05, for change from baseline assessment (paired *t*-test)

Compared with baseline assessments, a significant improvement (*p* < 0.05) in QOL (as assessed using EQ-5D) was observed at Week 104 in the overall cohort; however, this improvement (change from baseline) was not significant at Week 104 in any of the treatment groups. Additionally, the improvement in EQ-5D scores was not significantly different between the groups at Week 104 (Table 4).

A gradual improvement in the PANSS total score was observed over Week 52, which was sustained until Week 104 in all treatment groups (Online Resource 1 Fig. S1). At Week 104, a significant improvement from baseline in the total PANSS scores (including all subscales) was observed in all three treatment groups; however, no significant differences were observed between the groups at Week 104. For paliperidone, the improvement at Week 104 from Week 52 was significant for the total PANSS score and subscales such as positive symptoms and general psychopathology score; however, it did not contribute toward any significant difference between the treatment groups (Online Resource 1 Table S3).

The incidence of serious ADRs in the aripiprazole, blonanserin, and paliperidone groups was not significantly different (11.0%, 8.2%, and 11.9%, respectively) at Week 104; serious ADRs were marginally lower in the blonanserin group than in the other groups (Table 5). At Week 104, the most frequent serious ADRs in the aripiprazole, blonanserin, and paliperidone groups were psychiatric disorders (8.5%, 4.7%, and 8.3%, respectively), followed by nervous system disorders (2.4%, 1.2%, and 1.2%, respectively) (Table 5). One drug-related serious ADR, a case of completed suicide, was observed in the paliperidone group while on treatment for approximately 10 months. This patient was a 40-year-old woman with paranoid schizophrenia (DSM-IV-TR), a disease duration of 10 years and 8 months, and constipation as a complication.

**Table 5:** Incidence of serious ADRs up to Week 104

Item	All groups	Aripiprazole group	Blonanserin group	Paliperidone group	χ^2^ test (*p* value)
	*n* (%)	*n* (%)	*n* (%)	*n* (%)	
Number of patients, *N*	251	82	85	84	
Number of patients showing onset of serious ADRs	26 (10.4)	9 (11.0)	7 (8.2)	10 (11.9)	0.7180
Metabolism and nutrition disorders	3 (1.2)	0 (0.0)	1 (1.2)	2 (2.4)	0.3693
Diabetes mellitus	2 (0.8)	0 (0.0)	0 (0.0)	2 (2.4)	
Hyponatremia	1 (0.4)	0 (0.0)	1 (1.2)	0 (0.0)	
Psychiatric disorders	18 (7.2)	7 (8.5)	4 (4.7)	7 (8.3)	0.5554
Anxiety	1 (0.4)	0 (0.0)	0 (0.0)	1 (1.2)	
Completed suicide	1 (0.4)	0 (0.0)	0 (0.0)	1 (1.2)	
Delusion	1 (0.4)	1 (1.2)	0 (0.0)	0 (0.0)	
Hallucinations	1 (0.4)	1 (1.2)	0 (0.0)	0 (0.0)	
Auditory hallucination	1 (0.4)	1 (1.2)	0 (0.0)	0 (0.0)	
Schizophrenia	14 (5.6)	4 (4.9)	4 (4.7)	6 (7.1)	
Suicidal ideation	1 (0.4)	0 (0.0)	0 (0.0)	1 (1.2)	
Random	1 (0.4)	0 (0.0)	0 (0.0)	1 (1.2)	
Psychotic disorder	1 (0.4)	1 (1.2)	0 (0.0)	0 (0.0)	
Nervous system disorders	4 (1.6)	2 (2.4)	1 (1.2)	1 (1.2)	0.7576
Loss of consciousness	1 (0.4)	1 (1.2)	0 (0.0)	0 (0.0)	
Stuttering	2 (0.8)	1 (1.2)	0 (0.0)	1 (1.2)	
Delayed dyskinesia	1 (0.4)	0 (0.0)	1 (1.2)	0 (0.0)	
Musculoskeletal and connective tissue disorders	1 (0.4)	0 (0.0)	1 (1.2)	0 (0.0)	0.3752
Rhabdomyolysis	1 (0.4)	0 (0.0)	1 (1.2)	0 (0.0)	
Renal and urinary disorders	1 (0.4)	1 (1.2)	0 (0.0)	0 (0.0)	0.3554
Urinary retention	1 (0.4)	1 (1.2)	0 (0.0)	0 (0.0)	
Reproductive system and breast disorders	1 (0.4)	1 (1.2)	0 (0.0)	0 (0.0)	0.3554
Benign prostatic hyperplasia	1 (0.4)	1 (1.2)	0 (0.0)	0 (0.0)	
Injury, poisoning, and procedural Complications	1 (0.4)	1 (1.2)	0 (0.0)	0 (0.0)	0.3554
Issues of intentional product use	1 (0.4)	1 (1.2)	0 (0.0)	0 (0.0)	

*ADR* adverse drug reaction

Metabolic abnormality of diabetes mellitus as a serious ADR occurred in two patients in the paliperidone group (2.4%; occurred in one patient before Week 52 and in one patient after Week 52) but in none in the aripiprazole (0.0%) and blonanserin (0.0%) groups (Table 5) and was rated as a significant ADR. There was no significant difference in the CGI-SS scores in any treatment group and among the groups over 104 weeks (Kruskal-Wallis test; Online Resource 1 Tables S4 and S5). There was no significant difference in the CGI-I scores in any treatment group and among the groups over 104 weeks (Kruskal-Wallis test; Online Resource 1 Table S6). All AEs that occurred over 104 weeks in the treatment groups are presented in Online Resource 1 Table S7. There was no significant difference in the incidence of AEs between the groups over 104 weeks; however, the AE rate was marginally lower in the blonanserin group than in the other groups.

Metabolic and nutritional disorders were slightly more commonly observed in the blonanserin group (3.5%), followed by the paliperidone group (2.4%), compared with none in the aripiprazole group (0.0%; Online Resource 1 Table S7). There were no significant within-group or between-group differences in the DIEPSS score at Week 104 (Online Resource 1 Table S8).

## Discussion

JUMPs is a naturalistic study providing conclusive evidence over 104-week follow-up outcomes of three commonly used SGAs (aripiprazole, blonanserin, and paliperidone). Overall, our results showed that the trend observed for all endpoints remained constant from 52 weeks to 104 weeks, demonstrating no significant differences between the SGAs. Being a naturalistic study, the rates of treatment discontinuation and remission were considered the two key outcomes. The primary outcome was the rate of treatment discontinuation, which is a practical measure of real-world effectiveness and safety. Additionally, QOL and social functioning, which are important treatment effectiveness outcomes in daily clinical practice, were also evaluated.

We report an increase in treatment discontinuation rates from Week 52 (68.3% vs. 68.2% vs. 65.5%) to Week 104 (80.5% vs. 81.2% vs. 71.4%) in the aripiprazole, blonanserin, and paliperidone groups, respectively, which can be considered high. Regarding the reasons for discontinuations, some differences were observed between paliperidone and the other two drugs. The rates of discontinuation owing to inadequate efficacy were higher for the aripiprazole and blonanserin groups than for the paliperidone group at 52 weeks and 104 weeks. Paliperidone had slightly more discontinuation rates owing to poor tolerability or AEs up to 52 weeks than aripiprazole and blonanserin [11], but this rate decreased in cases that continued into the second year. This suggests that fewer cases of discontinuations due to AEs remained in the second year, and symptoms/tolerance tended to improve in cases that did continue in the second year. By contrast, aripiprazole and blonanserin showed a similar trend in discontinuation at 52 weeks and 104 weeks. The remission rates observed at Week 104 (aripiprazole, 42.9%; blonanserin, 46.7%; paliperidone, 45.8%) among patients who continued treatment were similar to those observed at Week 52 (aripiprazole, 42.3%; blonanserin, 40.7%; paliperidone, 41.4%); no differences in social functioning between the groups was observed, resulting in overall comparable outcomes. In the paliperidone group, a significant improvement was observed in the PANSS total score and subscale scores of positive symptoms and general psychopathology at Week 104 compared with those at Week 52.

Although direct comparisons cannot be made with the antipsychotic treatment landscape because of differences in formulations, drug classes, and study design, the treatment discontinuation rate at 104 weeks (range: 71.4–81.2%) in this study was similar to that at 18 months reported in the CATIE study (olanzapine [64%], ziprasidone [79%], perphenazine [75%], quetiapine [82%], and risperidone [74%]) [4]. Lower treatment discontinuation rates were reported in the SOHO study at 36 months (olanzapine [36%], risperidone [43%], quetiapine [69%], amisulpride [54%], clozapine [33%], oral typical antipsychotics [53%], and depot typical antipsychotics [51%]) and the EUFEST study over 52 weeks (haloperidol [72%], amisulpride [40%], olanzapine [33%], quetiapine [53%], and ziprasidone [45%]) [5, 7]. Long-acting injectables (LAIs) were designed to provide lower discontinuation rates/higher continuation rates and have been assessed in several studies using a similar naturalistic design or real-world evaluations. A naturalistic study with aripiprazole LAI reported a treatment discontinuation rate of 40.0% at Year 1 [23]. A 2-year naturalistic study with paliperidone 3-monthly LAI reported that 59.1% of patients continued the LAI and 69.4% were receiving some form of paliperidone LAI at the 2-year endpoint [24]. Another long-term study evaluated the real-world effectiveness of aripiprazole LAI in comparison with that of once-monthly paliperidone palmitate (PP1M). The results showed that 37% of patients receiving aripiprazole LAI and 34% of those receiving PP1M stopped treatment before or at 104 weeks [25]. The 2-year discontinuation rates of oral SGA in this study were higher (about twice as high) than those of LAI. However, the patients who select LAI treatment differ with regards to the presence or absence of previous oral medication therapy, its efficacy and tolerability, the state of disease stability in schizophrenia, and the patient’s habits and preferences for maintaining adherence. Furthermore, due to the nature of the dosage form, oral medications and LAIs differ in their ease/form of discontinuation (ease of transition to the next treatment). Therefore, long-term treatment retention rates should be evaluated with these considerations in mind.

Multivariable analysis identified that patients with a shorter duration of illness and a baseline CP-equivalent dose exceeding 1000 mg exhibited a higher rate of SGA treatment discontinuation. The CATIE study [4] identified higher baseline PANSS scores, younger age, longer duration of antipsychotic use, and antipsychotic medication taken before study enrollment as predictors of treatment interruption. In the current study, we found no association between treatment discontinuation and PANSS total score; however, these results may be affected by data analysis using a binary variable, namely, moderate and severe. Patients with a shorter duration of illness and a baseline CP-equivalent dose greater than 1000 mg had a higher rate of treatment discontinuation, which was somewhat different from the findings in the CATIE study [4] that identified a longer duration of antipsychotic use as a predictor of treatment discontinuation. This discrepancy in the findings is presumably because of differences in patient characteristics, such as the relatively younger age of patients in the CATIE study [4]. It was speculated that the time to treatment discontinuation would be shorter in patients with a shorter disease duration, based on the hypothesis that more patients were in the critical or acute period and, thus, at an increased risk of relapse [21, 26]. Achieving or maintaining remission is one of the key outcomes in schizophrenia, and maintaining mild symptoms for at least 6 months, as defined by Andreasen et al. [14], is a standard assessment parameter used in studies that evaluate the outcomes of schizophrenia treatment [27, 28]. In this study, the three agents were comparable in terms of discontinuation rates as well as remission rates. The trend of change in the PANSS score, including the remission rate observed in this study, is similar to that reported in previously published studies on oral antipsychotics [29–32].

Regarding safety, five patients had metabolic and nutritional abnormalities, including three with diabetes mellitus and one with dyslipidemia, and only four had weight gain; the incidence rates were lower than those observed in the CATIE and EUFEST studies [4, 5]. These safety results may have been due to differences in the drug profiles or may suggest fewer concerns about these metabolic changes in Japanese patients treated with SGAs.

Regarding limitations, the results of this study are dependent on the Japanese healthcare system environment and treatment policy at the time when the study was conducted and cannot be generalized for the current treatment landscape. Moreover, the result may have been influenced by the study protocol. For example, the CATIE study reported several drug options after treatment discontinuation, whereas in this study, only three choices of SGAs were recommended/available after treatment discontinuation at baseline. This may have influenced physician decisions regarding treatment discontinuation; hence, caution is required when comparing the findings of this study with those of other studies.

## Conclusion

The results of this 104-week naturalistic study showed that the rates of treatment discontinuation (range: 71–81%) and remission (range: 43–47%) were similar among Japanese patients treated with aripiprazole, blonanserin, and paliperidone. There were no differences in social functioning between groups, resulting in comparable outcomes. Among all patients, a shorter disease duration and a higher CP-equivalent antipsychotic dosage level (≥ 1000 mg) before switching to monotherapy were identified as factors affecting SGA treatment discontinuation.

## Electronic supplementary material

Below is the link to the electronic supplementary material.

Supplementary Material 1

## Data Availability

The datasets used and/or analyzed during the current study available from the corresponding author on reasonable request.
