# Supplementary material for: Randomized open-label study of second-generation antipsychotics for the treatment of schizophrenia: 104-week final results of the JUMPs study assessing treatment discontinuation, remission, and social functioning
Source: BMC Psychiatry. 2024 Sep 5;24:600. doi: 10.1186/s12888-024-06031-4 (PMC11376064; doi:10.1186/s12888-024-06031-4)
Supplement: Supplementary file 1 — Supplementary Material 1 [file 12888_2024_6031_MOESM1_ESM.docx]

**Supplementary information**

**Randomized open-label study of second-generation antipsychotics for the treatment of schizophrenia: 104-week final results of the JUMPs study assessing treatment discontinuation, remission, and social functioning**

**Authors:** Jun Ishigooka, Kazuyuki Nakagome, Tetsuro Ohmori, Nakao Iwata, Ken Inada, Jun-ichi Iga, Taro Kishi, Kiyoshi Fujita, Yuka Kikuchi, Toshiaki Shichijo, Hideaki Tabuse, Shotatsu Koretsune, Hiroshi Terada, Haruko Terada, Toshifumi Kishimoto, Yuichiro Tsutsumi, Kazutaka Ohi

**Corresponding author**

Jun Ishigooka, MD, PhD

Institute of CNS Pharmacology

4-26-11, Sendagaya

Shibuya-ku

Tokyo 151-0051

Japan

Cell phone: +81-90-8640-8182

Telephone: +81-3-6455-4173

Fax: +81-3-6455-4174

E-mail: ishigooka@i-cnsp.com

**Supplementary tables**

**Table S1** Use of concomitant medications

**Table S2** Rate of study discontinuation categorized by reasons

**Table S3** PANSS scores over 104 weeks

*max* maximum, *min* minimum, *PANSS* Positive and Negative Syndrome Scale, *SD* standard deviation

**Table S4** CGI-SS (Part 1)

*CGI-SS* Clinical Global Impression Scale for Severity of Suicidality

**Table S5** CGI-SS (Part 2)

*CGI-SS* Clinical Global Impression Scale for Severity of Suicidality

**Table S6** Overall improvement: CGI-I

*CGI-I* Clinical Global Impression Scale for Improvement

**Table S7** Incidence of AEs

*AE* adverse event

**Table S8** Drug-induced Extrapyramidal Symptoms Scale (DIEPSS) total scores

*max* maximum, *min* minimum, *SD* standard deviation

**Supplementary figure**

**Fig. S1** Change in PANSS total score

*PANSS* Positive and Negative Syndrome Scale, *SD* standard deviation

**Table S1** Use of concomitant medications

| Concomitant medications/training | Conditions |
| --- | --- |
| Antipsychotics | - Concomitant use of any antipsychotic other than the study drugs was prohibited. - If required, concomitant use of a single oral add-on antipsychotic was permitted at dosage levels within the approved range, for up to four doses in 4 weeks.   - The permitted antipsychotics include vegetamin, a combination therapy of chlorpromazine hydrochloride, promethazine hydrochloride, and phenobarbital. |
| Antianxiety drugs or antidepressants | - Antianxiety drugs or antidepressants used concomitantly before the start of protocol treatment were continued during the study period without changing the drugs or their dosage. However, initiating these drugs during the study period was not permitted. - If required, concomitant lorazepam was allowed (up to 10 doses in 4 weeks and at a daily dosage level within the approved range). |
| Anti-Parkinson drugs | - Anti-Parkinson drugs prescribed before baseline assessment were continued, and their dose was adjusted wherever applicable. Anti-Parkinson drugs were also allowed for the treatment of extrapyramidal symptoms but not as prophylactic therapy/prophylaxis. |
| Hypnotics | - The use of hypnotics was permitted for the treatment of insomnia during the study period. |
| Social skills training, occupational therapy, and psychotherapy | - There were no limitations set for therapies such as social skills training, occupational therapy, and psychotherapy. |

**Table S2** Rate of study discontinuation categorized by reasons

|  | Overall  (*N* = 251) | | Aripiprazole  (*n* = 82) | | Blonanserin  (*n* = 85) | | Paliperidone  (*n* = 84) | | χ^2^ test *p* value |
| --- | --- | --- | --- | --- | --- | --- | --- | --- | --- |
| Treatment discontinuation before or at Week 52 | 169 | (67.3) | 56 | (68.3) | 58 | (68.2) | 55 | (65.5) | 0.9060 |
| Reason for discontinuation |  |  |  |  |  |  |  |  |  |
| Inadequate efficacy | 75 | (29.9) | 28 | (34.1) | 28 | (32.9) | 19 | (22.6) | 0.2013 |
| Poorly tolerated or adverse events | 48 | (19.1) | 16 | (19.5) | 11 | (12.9) | 21 | (25.0) | 0.1364 |
| Patient’s request | 28 | (11.2) | 7 | (8.5) | 12 | (14.1) | 9 | (10.7) | 0.5126 |
| Reasons other than the above | 18 | (7.2) | 5 | (6.1) | 7 | (8.2) | 6 | (7.1) | 0.8665 |
| Treatment discontinuation by Week 104 (total) | 195 | (77.7) | 66 | (80.5) | 69 | (81.2) | 60 | (71.4) | 0.2385 |
| Reason for discontinuation |  |  |  |  |  |  |  |  |  |
| Inadequate efficacy | 86 | (34.3) | 34 | (41.5) | 30 | (35.3) | 22 | (26.2) | 0.1132 |
| Poorly tolerated or adverse events | 51 | (20.3) | 16 | (19.5) | 13 | (15.3) | 22 | (26.2) | 0.2073 |
| Patient’s request | 32 | (12.7) | 9 | (11.0) | 14 | (16.5) | 9 | (10.7) | 0.4487 |
| Reasons other than the above | 26 | (10.4) | 7 | (8.5) | 12 | (14.1) | 7 | (8.3) | 0.3757 |

**Table S3** PANSS scores over 104 weeks

| Item and treatment group | | | Baseline assessment | Week 12 | | Week 26 | | Week 52 | | Week 104 | | | |
| --- | --- | --- | --- | --- | --- | --- | --- | --- | --- | --- | --- | --- | --- |
|  |  |  |  | Score | Change | Score | Change | Score | Change | Score | Change from baseline | | Change from 52 weeks |
| Positive symptoms total | | |  |  |  |  |  |  |  |  |  | |  |
|  | Overall | *N* | 251 | 155 | 155 | 110 | 110 | 79 | 79 | 52 | 52 | | 50 |
|  |  | Mean (SD) | 15.9 (6.2) | 14.2 (5.9) | −1.4 (3.6)** | 13.7 (5.9) | −1.9 (3.8)** | 13.1 (5.7) | −1.9 (3.8)** | 12.6 (5.7) | −2.2 (3.7)** | | −0.3 (1.4) |
|  |  | Median | 15.0 | 13.0 | 0.0 | 12.0 | −1.0 | 12.0 | −1.0 | 11.0 | −1.0 | | 0.0 |
|  |  | Min, max | 7, 41 | 7, 36 | −17, 17 | 7, 39 | −23, 7 | 7, 29 | −17, 8 | 7, 32 | −17, 5 | | −4, 4 |
|  | Aripiprazole | *n* | 82 | 53 | 53 | 38 | 38 | 24 | 24 | 14 | 14 | | 12 |
|  | group | Mean (SD) | 16.0 (6.2) | 14.8 (6.6) | −1.5 (3.5)* | 14.6 (7.3) | −1.4 (3.2)* | 13.4 (7.0) | −2.2 (3.6)* | 13.4 (7.7) | −2.5 (3.0)* | | 0.2 (1.8) |
|  |  | Median | 15.0 | 13.0 | −1.0 | 12.0 | −1.0 | 11.0 | −2.5 | 10.5 | −2.5 | | 0.0 |
|  |  | Min, max | 7, 35 | 7, 36 | −9, 12 | 7, 39 | −7, 7 | 7, 29 | −9, 8 | 7, 32 | −7, 4 | | −2, 4 |
|  | Blonanserin | *n* | 85 | 49 | 49 | 36 | 36 | 27 | 27 | 15 | 15 | | 15 |
|  | group | Mean (SD) | 16.3 (5.7) | 14.6 (6.1) | −1.0 (3.8) | 13.3 (5.0) | −2.0 (3.1)* | 12.7 (4.3) | −2.2 (3.8)* | 13.3 (5.2) | −1.8 (2.8)** | | −0.2 (1.1) |
|  |  | Median | 16.0 | 14.0 | 0.0 | 12.5 | −1.0 | 12.0 | −1.0 | 14.0 | −2.0 | | 0.0 |
|  |  | Min, max | 7, 34 | 7, 31 | −9, 17 | 7, 28 | −10, 3 | 7, 28 | −15, 2 | 7, 28 | −8, 2 | | −4, 1 |
|  | Paliperidone | *n* | 84 | 53 | 53 | 36 | 36 | 28 | 28 | 23 | 23 | | 23 |
|  | group | Mean (SD) | 15.2 (6.7) | 13.1 (4.9) | −1.6 (3.5)* | 13.0 (5.1) | −2.4 (5.0)* | 13.2 (5.8) | −1.5 (4.1) | 11.7 (4.5) | −2.3 (4.6)* | | −0.6 (1.3)* |
|  |  | Median | 13.0 | 12.0 | 0.0 | 11.5 | −1.0 | 11.5 | −0.5 | 10.0 | −1.0 | | 0.0 |
|  |  | Min, max | 7, 41 | 7, 27 | −17, 3 | 7, 27 | −23, 3 | 7, 27 | −17, 4 | 7, 27 | −17, 5 | | −4, 2 |
| Negative symptoms total | | |  |  |  |  |  |  |  |  |  | |  |
|  | Overall | *N* | 251 | 155 | 155 | 110 | 110 | 79 | 79 | 52 | | 52 | 50 |
|  |  | Mean (SD) | 20.4 (6.3) | 18.9 (6.2) | −1.7 (3.9)** | 18.9 (6.8) | −2.0 (4.2)** | 18.5 (7.1) | −2.7 (5.0)** | 17.8 (7.4) | −3.3 (4.4)** | | −0.5 (2.1) |
|  |  | Median | 20.0 | 18.0 | −1.0 | 18.5 | −1.0 | 17.0 | −1.0 | 17.0 | −2.0 | | 0.0 |
|  |  | Min, max | 7, 43 | 7, 38 | −16, 13 | 7, 40 | −16, 11 | 7, 35 | −16, 8 | 7, 36 | −18, 4 | | −8, 4 |
|  | Aripiprazole | *n* | 82 | 53 | 53 | 38 | 38 | 24 | 24 | 14 | 14 | | 12 |
|  | group | Mean (SD) | 21.3 (6.4) | 18.8 (6.0) | −2.4 (4.7)* | 17.9 (6.5) | −2.8 (5.1)* | 17.8 (7.7) | −4.6 (6.3)*^,^ *** | 17.4 (8.4) | −4.2 (5.9)* | | −0.2 (2.0) |
|  |  | Median | 21.0 | 18.0 | −1.0 | 16.5 | −1.0 | 16.0 | −4.0 | 14.5 | −2.5 | | 0.0 |
|  |  | Min, max | 10, 36 | 8, 34 | −16, 13 | 7, 35 | −16, 11 | 9, 35 | −16, 8 | 7, 36 | −18, 4 | | −4, 4 |
|  | Blonanserin | *n* | 85 | 49 | 49 | 36 | 36 | 27 | 27 | 15 | 15 | | 15 |
|  | group | Mean (SD) | 20.7 (6.3) | 18.9 (7.2) | −1.7 (3.1)* | 19.8 (8.0) | −2.0 (3.3)* | 18.4 (6.8) | −2.7 (4.2)* | 18.7 (7.1) | −4.3 (3.3)** | | −0.4 (1.6) |
|  |  | Median | 20.0 | 18.0 | −1.0 | 20.0 | −1.0 | 18.0 | −2.0 | 19.0 | −5.0 | | 0.0 |
|  |  | Min, max | 9, 43 | 7, 38 | −13, 3 | 7, 40 | −9, 7 | 7, 30 | −13, 5 | 7, 30 | −9, 0 | | −3, 4 |
|  | Paliperidone | *n* | 84 | 53 | 53 | 36 | 36 | 28 | 28 | 23 | 23 | | 23 |
|  | group | Mean (SD) | 19.4 (6.1) | 19.0 (5.7) | −1.1 (3.5)* | 19.1 (5.9) | −1.2 (3.8) | 19.1 (6.9) | −1.0 (3.7) | 17.6 (7.2) | −2.1 (3.7)* | | −0.7 (2.5) |
|  |  | Median | 19.0 | 19.0 | 0.0 | 19.0 | 0.0 | 17.5 | 0.0 | 16.0 | −1.0 | | 0.0 |
|  |  | Min, max | 7, 33 | 7, 32 | −15, 9 | 7, 32 | −16, 7 | 7, 32 | −11, 8 | 7, 32 | −12, 4 | | −8, 4 |
| General psychopathology | | |  |  |  |  |  |  |  |  |  | |  |
|  | Overall | *N* | 251 | 155 | 155 | 110 | 110 | 79 | 79 | 52 | 52 | | 50 |
|  |  | Mean (SD) | 37.5 (11.0) | 34.2 (10.4) | −2.5 (6.7)** | 33.6 (10.9) | −3.4 (7.8)** | 32.6 (12.4) | −3.9 (8.2)** | 31.1 (12.0) | −5.2 (8.8)** | | −0.8 (3.3) |
|  |  | Median | 36.0 | 33.0 | −1.0 | 31.0 | −1.0 | 29.0 | −3.0 | 27.0 | −3.5 | | −0.5 |
|  |  | Min, max | 17, 80 | 16, 69 | −30, 19 | 16, 71 | −43, 21 | 16, 72 | −27, 22 | 16, 71 | −29, 21 | | −10, 11 |
|  | Aripiprazole | *n* | 82 | 53 | 53 | 38 | 38 | 24 | 24 | 14 | 14 | | 12 |
|  | group | Mean (SD) | 37.9 (11.8) | 34.4 (11.4) | −3.3 (7.2)* | 33.7 (12.8) | −3.1 (8.7)* | 32.1 (15.7) | −4.9 (10.1)* | 30.6 (16.0) | −6.6 (11.3)* | | −0.4 (3.9) |
|  |  | Median | 35.5 | 32.0 | −2.0 | 31.0 | −1.0 | 28.5 | −9.0 | 25.0 | −9.5 | | −1.0 |
|  |  | Min, max | 19, 80 | 16, 69 | −25, 19 | 17, 71 | −27, 21 | 16, 72 | −19, 22 | 16, 71 | −28, 21 | | −4, 11 |
|  | Blonanserin | *n* | 85 | 49 | 49 | 36 | 36 | 27 | 27 | 15 | 15 | | 15 |
|  | group | Mean (SD) | 38.7 (9.4) | 34.7 (10.8) | −2.7 (6.2)* | 34.3 (10.7) | −3.9 (6.5)* | 32.5 (10.9) | −5.2 (7.1)* | 33.3 (11.2) | −6.0 (7.9)* | | −0.2 (3.3) |
|  |  | Median | 38.0 | 34.0 | −1.0 | 32.0 | −2.0 | 31.0 | −4.0 | 34.0 | −2.0 | | 0.0 |
|  |  | Min, max | 18, 58 | 16, 63 | −24, 14 | 16, 58 | −21, 9 | 16, 58 | −27, 7 | 16, 58 | −20, 5 | | −6, 7 |
|  | Paliperidone | *n* | 84 | 53 | 53 | 36 | 36 | 28 | 28 | 23 | 23 | | 23 |
|  | group | Mean (SD) | 35.9 (11.7) | 33.5 (9.1) | −1.5 (6.7) | 33.0 (9.1) | −3.2 (8.3)* | 33.0 (10.9) | −1.7 (7.2) | 29.9 (9.8) | −3.8 (7.8)* | | −1.4 (2.9)* |
|  |  | Median | 33.0 | 33.0 | 0.0 | 30.0 | 0.0 | 29.0 | 0.0 | 27.0 | −2.0 | | −1.0 |
|  |  | Min, max | 17, 80 | 16, 53 | −30, 17 | 18, 50 | −43, 6 | 19, 59 | −25, 13 | 19, 50 | −29, 10 | | −10, 3 |
| PANSS score total | | |  |  |  |  |  |  |  |  |  | |  |
|  | Overall | *N* | 251 | 155 | 155 | 110 | 110 | 79 | 79 | 52 | 52 | | 50 |
|  |  | Mean (SD) | 73.8 (21.1) | 67.2 (20.4) | −5.6 (11.9)** | 66.2 (22.0) | −7.3 (13.8)** | 64.1 (23.6) | −8.5 (14.6)** | 61.5 (23.4) | −10.7 (14.4)** | | −1.6 (5.2)* |
|  |  | Median | 70.0 | 65.0 | −2.0 | 63.0 | −4.0 | 58.0 | −7.0 | 56.5 | −9.5 | | −1.0 |
|  |  | Min, max | 34, 147 | 30, 131 | −56, 29 | 30, 142 | −82, 35 | 30, 136 | −53, 26 | 30, 139 | −58, 25 | | −16, 19 |
|  | Aripiprazole | *n* | 82 | 53 | 53 | 38 | 38 | 24 | 24 | 14 | 14 | | 12 |
|  | group | Mean (SD) | 75.2 (22.2) | 68.0 (22.3) | −7.2 (12.8)* | 66.2 (25.3) | −7.3 (15.0)* | 63.3 (29.6) | −11.6 (17.7)* | 61.4 (31.2) | −13.4 (17.6)* | | −0.4 (7.0) |
|  |  | Median | 72.0 | 66.0 | −4.0 | 60.0 | −4.0 | 54.0 | −17.0 | 51.0 | −14.5 | | −1.0 |
|  |  | Min, max | 37, 147 | 31, 131 | −31, 25 | 31, 142 | −40, 35 | 33, 136 | −39, 26 | 30, 139 | −37, 25 | | −9, 19 |
|  | Blonanserin | *n* | 85 | 49 | 49 | 36 | 36 | 27 | 27 | 15 | 15 | | 15 |
|  | group | Mean (SD) | 75.7 (18.4) | 68.2 (21.3) | −5.3 (11.1)* | 67.3 (22.2) | −7.9 (10.7)** | 63.6 (20.2) | −10.1 (12.2)* | 65.2 (21.2) | −12.1 (11.1)** | | −0.8 (4.8) |
|  |  | Median | 76.0 | 66.0 | −2.0 | 66.0 | −7.0 | 63.0 | −7.0 | 64.0 | −8.0 | | 0.0 |
|  |  | Min, max | 36, 119 | 31, 121 | −40, 29 | 30, 121 | −38, 14 | 30, 116 | −41, 7 | 31, 116 | −34, 0 | | −7, 11 |
|  | Paliperidone | *n* | 84 | 53 | 53 | 36 | 36 | 28 | 28 | 23 | 23 | | 23 |
|  | group | Mean (SD) | 70.5 (22.4) | 65.6 (17.6) | −4.2 (11.9)* | 65.1 (18.2) | −6.8 (15.4)* | 65.3 (21.6) | −4.3 (13.2) | 59.1 (19.9) | −8.3 (14.3)* | | −2.7 (4.4)* |
|  |  | Median | 65.0 | 65.0 | −1.0 | 63.0 | −2.0 | 61.5 | −3.0 | 57.0 | −5.0 | | −2.0 |
|  |  | Min, max | 34, 147 | 30, 106 | −56, 20 | 34, 106 | −82, 10 | 34, 117 | −53, 24 | 33, 109 | −58, 17 | | −16, 4 |

*max* maximum, *min* minimum, *PANSS* Positive and Negative Syndrome Scale, *SD* standard deviation

**p* < 0.05 and ***p* < 0.0001 for change from baseline assessment (paired *t*-test)

****p* = 0.034 for improvement in negative symptoms between groups at Week 52 (analysis of variance)

**Table S4** CGI-SS (Part 1)

| Treatment group | Grade | Baseline assessment | Week 12 | Week 26 | Week 52 | Week 104 |
| --- | --- | --- | --- | --- | --- | --- |
|  |  | *n* (%) | *n* (%) | *n* (%) | *n* (%) | *n* (%) |
| Overall | Normal | 242 (96.4) | 149 (98.7) | 103 (96.3) | 75 (96.2) | 52 (100.0) |
|  | Mild suicidality | 9 (3.6) | 2 (1.3) | 4 (3.7) | 2 (2.6) | 0 (0.0) |
|  | Moderate suicidality | 0 (0.0) | 0 (0.0) | 0 (0.0) | 1 (1.3) | 0 (0.0) |
|  | Severe suicidality | 0 (0.0) | 0 (0.0) | 0 (0.0) | 0 (0.0) | 0 (0.0) |
|  | Attempted suicide | 0 (0.0) | 0 (0.0) | 0 (0.0) | 0 (0.0) | 0 (0.0) |
| Aripiprazole group | Normal | 80 (97.6) | 50 (98.0) | 35 (97.2) | 25 (96.2) | 13 (100.0) |
|  | Mild suicidality | 2 (2.4) | 1 (2.0) | 1 (2.8) | 1 (3.8) | 0 (0.0) |
|  | Moderate suicidality | 0 (0.0) | 0 (0.0) | 0 (0.0) | 0 (0.0) | 0 (0.0) |
|  | Severe suicidality | 0 (0.0) | 0 (0.0) | 0 (0.0) | 0 (0.0) | 0 (0.0) |
|  | Attempted suicide | 0 (0.0) | 0 (0.0) | 0 (0.0) | 0 (0.0) | 0 (0.0) |
| Blonanserin group | Normal | 82 (96.5) | 46 (97.9) | 33 (94.3) | 23 (92.0) | 15 (100.0) |
|  | Mild suicidality | 3 (3.5) | 1 (2.1) | 2 (5.7) | 1 (4.0) | 0 (0.0) |
|  | Moderate suicidality | 0 (0.0) | 0 (0.0) | 0 (0.0) | 1 (4.0) | 0 (0.0) |
|  | Severe suicidality | 0 (0.0) | 0 (0.0) | 0 (0.0) | 0 (0.0) | 0 (0.0) |
|  | Attempted suicide | 0 (0.0) | 0 (0.0) | 0 (0.0) | 0 (0.0) | 0 (0.0) |
| Paliperidone group | Normal | 80 (95.2) | 53 (100.0) | 35 (97.2) | 27 (100.0) | 24 (100.0) |
|  | Mild suicidality | 4 (4.8) | 0 (0.0) | 1 (2.8) | 0 (0.0) | 0 (0.0) |
|  | Moderate suicidality | 0 (0.0) | 0 (0.0) | 0 (0.0) | 0 (0.0) | 0 (0.0) |
|  | Severe suicidality | 0 (0.0) | 0 (0.0) | 0 (0.0) | 0 (0.0) | 0 (0.0) |
|  | Attempted suicide | 0 (0.0) | 0 (0.0) | 0 (0.0) | 0 (0.0) | 0 (0.0) |

There was no significant difference over 52 weeks between the groups (Kruskal-Wallis test)

*CGI-SS* Clinical Global Impression Scale for Severity of Suicidality

**Table S5** CGI-SS (Part 2)

| Treatment group | Grade | Week 12 | Week 26 | Week 52 | Week 104 |
| --- | --- | --- | --- | --- | --- |
|  |  | *n* (%) | *n* (%) | *n* (%) | *n* (%) |
| Overall | Marked improvement | 2 (1.3) | 1 (0.9) | 4 (5.1) | 5 (9.6) |
|  | Moderate improvement | 9 (6.0) | 9 (8.4) | 2 (2.6) | 1 (1.9) |
|  | Minimal improvement | 11 (7.3) | 3 (2.8) | 3 (3.8) | 4 (7.7) |
|  | No change | 126 (83.4) | 91 (85.0) | 67 (85.9) | 42 (80.8) |
|  | Mild worsening | 2 (1.3) | 3 (2.8) | 1 (1.3) | 0 (0.0) |
|  | Moderate worsening | 1 (0.7) | 0 (0.0) | 1 (1.3) | 0 (0.0) |
|  | Marked worsening | 0 (0.0) | 0 (0.0) | 0 (0.0) | 0 (0.0) |
| Aripiprazole group | Marked improvement | 1 (2.0) | 0 (0.0) | 1 (3.8) | 2 (15.4) |
|  | Moderate improvement | 3 (5.9) | 3 (8.3) | 0 (0.0) | 0 (0.0) |
|  | Minimal improvement | 4 (7.8) | 1 (2.8) | 1 (3.8) | 2 (15.4) |
|  | No change | 42 (82.4) | 31 (86.1) | 24 (92.3) | 9 (69.2) |
|  | Mild worsening | 1 (2.0) | 1 (2.8) | 0 (0.0) | 0 (0.0) |
|  | Moderate worsening | 0 (0.0) | 0 (0.0) | 0 (0.0) | 0 (0.0) |
|  | Marked worsening | 0 (0.0) | 0 (0.0) | 0 (0.0) | 0 (0.0) |
| Blonanserin group | Marked improvement | 1 (2.1) | 0 (0.0) | 0 (0.0) | 1 (6.7) |
|  | Moderate improvement | 5 (10.6) | 5 (14.3) | 2 (8.0) | 1 (6.7) |
|  | Minimal improvement | 4 (8.5) | 2 (5.7) | 2 (8.0) | 1 (6.7) |
|  | No change | 35 (74.5) | 27 (77.1) | 20 (80.0) | 12 (80.0) |
|  | Mild worsening | 1 (2.1) | 1 (2.9) | 0 (0.0) | 0 (0.0) |
|  | Moderate worsening | 1 (2.1) | 0 (0.0) | 1 (4.0) | 0 (0.0) |
|  | Marked worsening | 0 (0.0) | 0 (0.0) | 0 (0.0) | 0 (0.0) |
| Paliperidone group | Marked improvement | 0 (0.0) | 1 (2.8) | 3 (11.1) | 2 (8.3) |
|  | Moderate improvement | 1 (1.9) | 1 (2.8) | 0 (0.0) | 0 (0.0) |
|  | Minimal improvement | 3 (5.7) | 0 (0.0) | 0 (0.0) | 1 (4.2) |
|  | No change | 49 (92.5) | 33 (91.7) | 23 (85.2) | 21 (87.5) |
|  | Mild worsening | 0 (0.0) | 1 (2.8) | 1 (3.7) | 0 (0.0) |
|  | Moderate worsening | 0 (0.0) | 0 (0.0) | 0 (0.0) | 0 (0.0) |
|  | Marked worsening | 0 (0.0) | 0 (0.0) | 0 (0.0) | 0 (0.0) |

*CGI-SS* Clinical Global Impression Scale for Severity of Suicidality

**Table S6** Overall improvement: CGI-I

| Treatment group | Grade | Week 12 | Week 26 | Week 52 | Week 104 |
| --- | --- | --- | --- | --- | --- |
|  |  | *n* (%) | *n* (%) | *n* (%) | *n* (%) |
| Overall | Marked improvement | 3 (2.0) | 4 (3.7) | 6 (7.7) | 7 (13.5) |
|  | Moderate improvement | 14 (9.3) | 18 (16.8) | 10 (12.8) | 7 (13.5) |
|  | Minimal improvement | 39 (25.8) | 15 (14.0) | 12 (15.4) | 7 (13.5) |
|  | No change | 82 (54.3) | 65 (60.7) | 46 (59.0) | 30 (57.7) |
|  | Mild worsening | 8 (5.3) | 5 (4.7) | 3 (3.8) | 1 (1.9) |
|  | Moderate worsening | 5 (3.3) | 0 (0.0) | 1 (1.3) | 0 (0.0) |
|  | Marked worsening | 0 (0.0) | 0 (0.0) | 0 (0.0) | 0 (0.0) |
| Aripiprazole group | Marked improvement | 2 (3.9) | 3 (8.3) | 4 (15.4) | 4 (30.8) |
|  | Moderate improvement | 7 (13.7) | 4 (11.1) | 1 (3.8) | 0 (0.0) |
|  | Minimal improvement | 10 (19.6) | 5 (13.9) | 3 (11.5) | 3 (23.1) |
|  | No change | 26 (51.0) | 21 (58.3) | 17 (65.4) | 6 (46.2) |
|  | Mild worsening | 3 (5.9) | 3 (8.3) | 1 (3.8) | 0 (0.0) |
|  | Moderate worsening | 3 (5.9) | 0 (0.0) | 0 (0.0) | 0 (0.0) |
|  | Marked worsening | 0 (0.0) | 0 (0.0) | 0 (0.0) | 0 (0.0) |
| Blonanserin group | Marked improvement | 1 (2.1) | 0 (0.0) | 0 (0.0) | 1 (6.7) |
|  | Moderate improvement | 4 (8.5) | 8 (22.9) | 7 (28.0) | 5 (33.3) |
|  | Minimal improvement | 15 (31.9) | 6 (17.1) | 6 (24.0) | 2 (13.3) |
|  | No change | 22 (46.8) | 19 (54.3) | 11 (44.0) | 7 (46.7) |
|  | Mild worsening | 3 (6.4) | 2 (5.7) | 0 (0.0) | 0 (0.0) |
|  | Moderate worsening | 2 (4.3) | 0 (0.0) | 1 (4.0) | 0 (0.0) |
|  | Marked worsening | 0 (0.0) | 0 (0.0) | 0 (0.0) | 0 (0.0) |
| Paliperidone group | Marked improvement | 0 (0.0) | 1 (2.8) | 2 (7.4) | 2 (8.3) |
|  | Moderate improvement | 3 (5.7) | 6 (16.7) | 2 (7.4) | 2 (8.3) |
|  | Minimal improvement | 14 (26.4) | 4 (11.1) | 3 (11.1) | 2 (8.3) |
|  | No change | 34 (64.2) | 25 (69.4) | 18 (66.7) | 17 (70.8) |
|  | Mild worsening | 2 (3.8) | 0 (0.0) | 2 (7.4) | 1 (4.2) |
|  | Moderate worsening | 0 (0.0) | 0 (0.0) | 0 (0.0) | 0 (0.0) |
|  | Marked worsening | 0 (0.0) | 0 (0.0) | 0 (0.0) | 0 (0.0) |

There was no significant difference over 52 weeks between the groups (Kruskal-Wallis test)

*CGI-I* Clinical Global Impression Scale for Improvement

**Table S7** Incidence of AEs

| Item | | Overall | Aripiprazole group | Blonanserin group | Paliperidone group | χ^2^ test *p* value |
| --- | --- | --- | --- | --- | --- | --- |
|  |  | *N* (%) | *n* (%) | *n* (%) | *n* (%) |  |
| Number of subjects | | 251 | 82 | 85 | 84 |  |
| Number of subjects having onset of AEs | | 135 (53.8) | 43 (52.4) | 40 (47.1) | 52 (61.9) | 0.1470 |
| Infections and infestations | | 14 (5.6) | 3 (3.7) | 4 (4.7) | 7 (8.3) | 0.3853 |
|  | Bronchitis | 1 (0.4) | 0 (0.0) | 0 (0.0) | 1 (1.2) |  |
|  | Diabetic gangrene | 1 (0.4) | 0 (0.0) | 0 (0.0) | 1 (1.2) |  |
|  | Gastroenteritis | 1 (0.4) | 0 (0.0) | 0 (0.0) | 1 (1.2) |  |
|  | Influenza | 4 (1.6) | 1 (1.2) | 1 (1.2) | 2 (2.4) |  |
|  | Mastitis | 1 (0.4) | 0 (0.0) | 0 (0.0) | 1 (1.2) |  |
|  | Nasopharyngitis | 7 (2.8) | 1 (1.2) | 3 (3.5) | 3 (3.6) |  |
|  | Salpingitis | 1 (0.4) | 0 (0.0) | 0 (0.0) | 1 (1.2) |  |
|  | Candidal infection | 1 (0.4) | 1 (1.2) | 0 (0.0) | 0 (0.0) |  |
| Neoplasms benign, malignant, and unspecified (including cysts and polyps) | | 2 (0.8) | 0 (0.0) | 2 (2.4) | 0 (0.0) | 0.1396 |
|  | Breast cancer | 1 (0.4) | 0 (0.0) | 1 (1.2) | 0 (0.0) |  |
|  | Gastric cancer | 1 (0.4) | 0 (0.0) | 1 (1.2) | 0 (0.0) |  |
| Endocrine disorders | | 9 (3.6) | 1 (1.2) | 0 (0.0) | 8 (9.5) | 0.0015 |
|  | Hyperprolactinemia | 8 (3.2) | 0 (0.0) | 0 (0.0) | 8 (9.5) |  |
|  | Hypoprolactinemia | 1 (0.4) | 1 (1.2) | 0 (0.0) | 0 (0.0) |  |
| Metabolism and nutrition disorders | | 5 (2.0) | 0 (0.0) | 3 (3.5) | 2 (2.4) | 0.2515 |
|  | Diabetes mellitus | 3 (1.2) | 0 (0.0) | 1 (1.2) | 2 (2.4) |  |
|  | Hypokalemia | 1 (0.4) | 0 (0.0) | 1 (1.2) | 0 (0.0) |  |
|  | Hyponatremia | 1 (0.4) | 0 (0.0) | 1 (1.2) | 0 (0.0) |  |
|  | Dyslipidemia | 1 (0.4) | 0 (0.0) | 1 (1.2) | 0 (0.0) |  |
|  | Decreased appetite | 1 (0.4) | 0 (0.0) | 1 (1.2) | 0 (0.0) |  |
| Psychiatric disorders | | 62 (24.7) | 22 (26.8) | 20 (23.5) | 20 (23.8) | 0.8614 |
|  | Aggression | 1 (0.4) | 0 (0.0) | 1 (1.2) | 0 (0.0) |  |
|  | Anger | 1 (0.4) | 0 (0.0) | 0 (0.0) | 1 (1.2) |  |
|  | Anxiety | 3 (1.2) | 1 (1.2) | 0 (0.0) | 2 (2.4) |  |
|  | Completed suicide | 2 (0.8) | 1 (1.2) | 0 (0.0) | 1 (1.2) |  |
|  | Delusion | 1 (0.4) | 1 (1.2) | 0 (0.0) | 0 (0.0) |  |
|  | Disorientation | 1 (0.4) | 1 (1.2) | 0 (0.0) | 0 (0.0) |  |
|  | Hallucinations | 1 (0.4) | 1 (1.2) | 0 (0.0) | 0 (0.0) |  |
|  | Auditory hallucination | 1 (0.4) | 1 (1.2) | 0 (0.0) | 0 (0.0) |  |
|  | Hypomania | 1 (0.4) | 1 (1.2) | 0 (0.0) | 0 (0.0) |  |
|  | Insomnia | 29 (11.6) | 9 (11.0) | 12 (14.1) | 8 (9.5) |  |
|  | Irritability | 1 (0.4) | 1 (1.2) | 0 (0.0) | 0 (0.0) |  |
|  | Restlessness | 1 (0.4) | 0 (0.0) | 0 (0.0) | 1 (1.2) |  |
|  | Schizophrenia | 20 (8.0) | 6 (7.3) | 6 (7.1) | 8 (9.5) |  |
|  | Suicidal ideation | 1 (0.4) | 0 (0.0) | 0 (0.0) | 1 (1.2) |  |
|  | Random | 1 (0.4) | 0 (0.0) | 0 (0.0) | 1 (1.2) |  |
|  | Psychotic disorder | 2 (0.8) | 1 (1.2) | 1 (1.2) | 0 (0.0) |  |
| Nervous system disorders | | 42 (16.7) | 14 (17.1) | 11 (12.9) | 17 (20.2) | 0.4438 |
|  | Akathisia | 20 (8.0) | 6 (7.3) | 5 (5.9) | 9 (10.7) |  |
|  | Disturbance in attention | 1 (0.4) | 0 (0.0) | 0 (0.0) | 1 (1.2) |  |
|  | Dizziness | 2 (0.8) | 0 (0.0) | 0 (0.0) | 2 (2.4) |  |
|  | Dyskinesia | 1 (0.4) | 1 (1.2) | 0 (0.0) | 0 (0.0) |  |
|  | Dystonia | 1 (0.4) | 0 (0.0) | 0 (0.0) | 1 (1.2) |  |
|  | Extrapyramidal disorders | 5 (2.0) | 1 (1.2) | 1 (1.2) | 3 (3.6) |  |
|  | Headache | 2 (0.8) | 0 (0.0) | 1 (1.2) | 1 (1.2) |  |
|  | Loss of consciousness | 1 (0.4) | 1 (1.2) | 0 (0.0) | 0 (0.0) |  |
|  | Oculogyric crises | 2 (0.8) | 1 (1.2) | 1 (1.2) | 0 (0.0) |  |
|  | Parkinsonism | 2 (0.8) | 2 (2.4) | 0 (0.0) | 0 (0.0) |  |
|  | Somnolence | 3 (1.2) | 2 (2.4) | 0 (0.0) | 1 (1.2) |  |
|  | Stuttering | 2 (0.8) | 1 (1.2) | 0 (0.0) | 1 (1.2) |  |
|  | Delayed dyskinesia | 1 (0.4) | 0 (0.0) | 1 (1.2) | 0 (0.0) |  |
|  | Tremor | 2 (0.8) | 0 (0.0) | 1 (1.2) | 1 (1.2) |  |
|  | Restless legs syndrome | 1 (0.4) | 0 (0.0) | 1 (1.2) | 0 (0.0) |  |
| Eye disorders | | 2 (0.8) | 0 (0.0) | 1 (1.2) | 1 (1.2) | 0.6131 |
|  | Cataract | 1 (0.4) | 0 (0.0) | 0 (0.0) | 1 (1.2) |  |
|  | Conjunctivitis | 1 (0.4) | 0 (0.0) | 1 (1.2) | 0 (0.0) |  |
| Cardiac disorders | | 2 (0.8) | 1 (1.2) | 0 (0.0) | 1 (1.2) | 0.5967 |
|  | Right bundle branch block | 1 (0.4) | 1 (1.2) | 0 (0.0) | 0 (0.0) |  |
|  | Palpitations | 1 (0.4) | 0 (0.0) | 0 (0.0) | 1 (1.2) |  |
| Vascular disorders | | 4 (1.6) | 0 (0.0) | 2 (2.4) | 2 (2.4) | 0.3730 |
|  | Hypertension | 4 (1.6) | 0 (0.0) | 2 (2.4) | 2 (2.4) |  |
| Respiratory, thoracic, and mediastinal disorders | | 1 (0.4) | 0 (0.0) | 0 (0.0) | 1 (1.2) | 0.3686 |
|  | Upper respiratory tract inflammation | 1 (0.4) | 0 (0.0) | 0 (0.0) | 1 (1.2) |  |
| Gastrointestinal disorders | | 22 (8.8) | 10 (12.2) | 5 (5.9) | 7 (8.3) | 0.3483 |
|  | Abdominal distension | 1 (0.4) | 0 (0.0) | 1 (1.2) | 0 (0.0) |  |
|  | Abdominal pain | 1 (0.4) | 0 (0.0) | 0 (0.0) | 1 (1.2) |  |
|  | Constipation | 6 (2.4) | 2 (2.4) | 2 (2.4) | 2 (2.4) |  |
|  | Diarrhea | 2 (0.8) | 0 (0.0) | 1 (1.2) | 1 (1.2) |  |
|  | Gastric ulcer | 1 (0.4) | 1 (1.2) | 0 (0.0) | 0 (0.0) |  |
|  | Nausea | 8 (3.2) | 5 (6.1) | 0 (0.0) | 3 (3.6) |  |
|  | Gum disease | 1 (0.4) | 1 (1.2) | 0 (0.0) | 0 (0.0) |  |
|  | Vomiting | 3 (1.2) | 2 (2.4) | 1 (1.2) | 0 (0.0) |  |
| Hepatobiliary disorders | | 1 (0.4) | 1 (1.2) | 0 (0.0) | 0 (0.0) | 0.3554 |
|  | Cholelithiasis | 1 (0.4) | 1 (1.2) | 0 (0.0) | 0 (0.0) |  |
| Skin and subcutaneous tissue disorders | | 3 (1.2) | 0 (0.0) | 1 (1.2) | 2 (2.4) | 0.3693 |
|  | Eczema | 2 (0.8) | 0 (0.0) | 1 (1.2) | 1 (1.2) |  |
|  | Rash | 1 (0.4) | 0 (0.0) | 0 (0.0) | 1 (1.2) |  |
| Musculoskeletal and connective tissue disorders | | 3 (1.2) | 0 (0.0) | 2 (2.4) | 1 (1.2) | 0.3759 |
|  | Arthralgia | 1 (0.4) | 0 (0.0) | 1 (1.2) | 0 (0.0) |  |
|  | Back pain | 1 (0.4) | 0 (0.0) | 0 (0.0) | 1 (1.2) |  |
|  | Pain in extremity | 1 (0.4) | 0 (0.0) | 1 (1.2) | 0 (0.0) |  |
|  | Rhabdomyolysis | 1 (0.4) | 0 (0.0) | 1 (1.2) | 0 (0.0) |  |
| Renal and urinary disorders | | 2 (0.8) | 1 (1.2) | 1 (1.2) | 0 (0.0) | 0.6020 |
|  | Dysuria | 1 (0.4) | 0 (0.0) | 1 (1.2) | 0 (0.0) |  |
|  | Urinary retention | 1 (0.4) | 1 (1.2) | 0 (0.0) | 0 (0.0) |  |
| Reproductive system and breast disorders | | 3 (1.2) | 1 (1.2) | 0 (0.0) | 2 (2.4) | 0.3626 |
|  | Amenorrhea | 1 (0.4) | 0 (0.0) | 0 (0.0) | 1 (1.2) |  |
|  | Benign prostatic hyperplasia | 1 (0.4) | 1 (1.2) | 0 (0.0) | 0 (0.0) |  |
|  | Endometriosis | 1 (0.4) | 0 (0.0) | 0 (0.0) | 1 (1.2) |  |
| General disorders and administration site conditions | | 4 (1.6) | 0 (0.0) | 4 (4.7) | 0 (0.0) | 0.0189 |
|  | Feeling abnormal | 1 (0.4) | 0 (0.0) | 1 (1.2) | 0 (0.0) |  |
|  | Irritability | 1 (0.4) | 0 (0.0) | 1 (1.2) | 0 (0.0) |  |
|  | Malaise | 1 (0.4) | 0 (0.0) | 1 (1.2) | 0 (0.0) |  |
|  | Dry mouth | 1 (0.4) | 0 (0.0) | 1 (1.2) | 0 (0.0) |  |
| Laboratory tests | | 14 (5.6) | 4 (4.9) | 4 (4.7) | 6 (7.1) | 0.7447 |
|  | Blood creatine phosphokinase increased | 2 (0.8) | 0 (0.0) | 1 (1.2) | 1 (1.2) |  |
|  | Blood glucose increased | 2 (0.8) | 1 (1.2) | 1 (1.2) | 0 (0.0) |  |
|  | Blood insulin abnormal | 1 (0.4) | 0 (0.0) | 1 (1.2) | 0 (0.0) |  |
|  | Blood prolactin increased | 4 (1.6) | 0 (0.0) | 1 (1.2) | 3 (3.6) |  |
|  | Electrocardiogram abnormal | 1 (0.4) | 0 (0.0) | 0 (0.0) | 1 (1.2) |  |
|  | Glycosylated hemoglobin increased | 1 (0.4) | 1 (1.2) | 0 (0.0) | 0 (0.0) |  |
|  | Weight gain | 4 (1.6) | 2 (2.4) | 0 (0.0) | 2 (2.4) |  |
|  | White blood cell count increased | 1 (0.4) | 0 (0.0) | 1 (1.2) | 0 (0.0) |  |
| Injury, poisoning, and procedural complications | | 5 (2.0) | 5 (6.1) | 0 (0.0) | 0 (0.0) | 0.0052 |
|  | Animal bite | 1 (0.4) | 1 (1.2) | 0 (0.0) | 0 (0.0) |  |
|  | Fracture of fibula | 1 (0.4) | 1 (1.2) | 0 (0.0) | 0 (0.0) |  |
|  | Tibial fracture | 1 (0.4) | 1 (1.2) | 0 (0.0) | 0 (0.0) |  |
|  | Contusion | 1 (0.4) | 1 (1.2) | 0 (0.0) | 0 (0.0) |  |
|  | Burns | 1 (0.4) | 1 (1.2) | 0 (0.0) | 0 (0.0) |  |
|  | Issues of intentional product use | 1 (0.4) | 1 (1.2) | 0 (0.0) | 0 (0.0) |  |

*AE* adverse event

**Table S8** Drug-induced Extrapyramidal Symptoms Scale (DIEPSS) total scores

|  | |  | Week 12 | | Week 26 | | Week 52 | | Week 104 | | |
| --- | --- | --- | --- | --- | --- | --- | --- | --- | --- | --- | --- |
|  |  |  | Score | Change | Score | Change | Score | Change | Score | Change from baseline | Change from 52 weeks |
| Overall | *N* | 251 | 154 | 154 | 110 | 110 | 80 | 80 | 52 | 52 | 52 |
|  | Mean (SD) | 1.5 (2.5) | 1.2 (1.8) | −0.4 (2.0) | 1.3 (2.1) | −0.3 (1.9) | 1.2 (2.1) | −0.2 (2.0) | 1.2 (2.2) | −0.1 (1.8) | 0.0 (1.0) |
|  | Median | 0.0 | 0.0 | 0.0 | 0.0 | 0.0 | 0.0 | 0.0 | 0.0 | 0.0 | 0.0 |
|  | Min, max | 0, 13 | 0, 10 | −9, 6 | 0, 10 | −9, 4 | 0, 9 | −10, 6 | 0, 9 | −7, 5 | −3, 3 |
| Aripiprazole | *n* | 82 | 53 | 53 | 38 | 38 | 25 | 25 | 13 | 13 | 13 |
| group | Mean (SD) | 2.1 (2.9) | 1.3 (1.8) | −0.6 (2.6) | 1.4 (2.0) | −0.2 (2.5) | 1.2 (1.8) | −0.1 (2.8) | 1.5 (2.0) | 0.2 (2.7) | 0.2 (1.4) |
|  | Median | 1.0 | 1.0 | 0.0 | 0.5 | 0.0 | 0.0 | 0.0 | 0.0 | 0.0 | 0.0 |
|  | Min, max | 0, 12 | 0, 7 | −9, 6 | 0, 6 | −9, 4 | 0, 6 | −10, 6 | 0, 5 | −7, 5 | −2, 3 |
| Blonanserin | *n* | 85 | 47 | 47 | 36 | 36 | 27 | 27 | 15 | 15 | 15 |
| group | Mean (SD) | 1.4 (2.5) | 1.0 (1.9) | −0.4 (1.6) | 1.5 (2.3) | −0.2 (1.4) | 1.9 (2.9) | 0.1 (1.7) | 2.3 (3.2) | 0.1 (1.9) | 0.1 (0.6) |
|  | Median | 0.0 | 0.0 | 0.0 | 0.5 | 0.0 | 0.0 | 0.0 | 1.0 | 0.0 | 0.0 |
|  | Min, max | 0, 12 | 0, 8 | −9, 2 | 0, 8 | −5, 3 | 0, 9 | −3, 5 | 0, 9 | −3, 5 | −1, 1 |
| Paliperidone | *n* | 84 | 54 | 54 | 36 | 36 | 28 | 28 | 24 | 24 | 24 |
| group | Mean (SD) | 1.1 (2.1) | 1.1 (1.8) | −0.1 (1.5) | 1.1 (2.0) | −0.4 (1.5) | 0.5 (1.0) | −0.5 (1.2) | 0.3 (0.7) | −0.4 (1.1) | −0.1 (0.9) |
|  | Median | 0.0 | 0.0 | 0.0 | 0.0 | 0.0 | 0.0 | 0.0 | 0.0 | 0.0 | 0.0 |
|  | Min, max | 0, 13 | 0, 10 | −6, 3 | 0, 10 | −5, 2 | 0, 3 | −3, 2 | 0, 2 | −4, 2 | −3, 2 |

*max* maximum, *min* minimum, *SD* standard deviation

**Fig. S1** Mean (SD) change in the PANSS total score


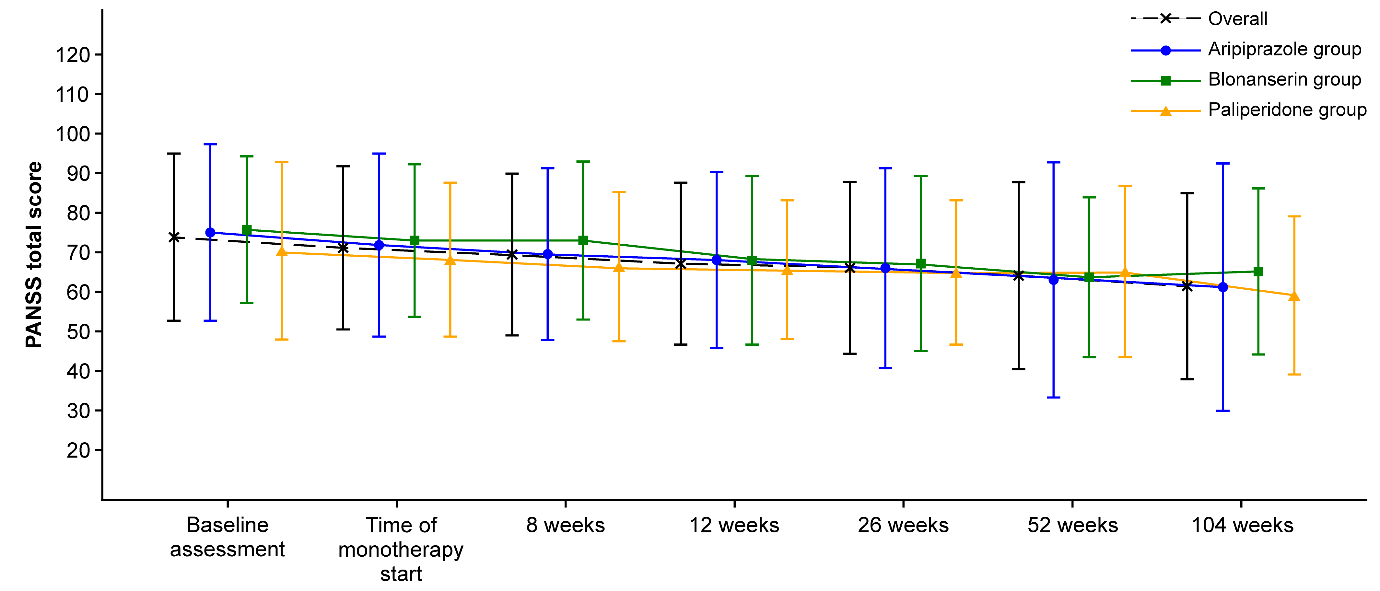


There was no significant between-group difference for any time point (*p* < 0.05)

*PANSS* Positive and Negative Syndrome Scale, *SD* standard deviation
